# The transcriptional regulator CBX2 and ovarian function: A whole genome and whole transcriptome approach

**DOI:** 10.1038/s41598-019-53370-4

**Published:** 2019-11-19

**Authors:** Leila Bouazzi, Patrick Sproll, Wassim Eid, Anna Biason-Lauber

**Affiliations:** 1 Division of Endocrinology, Section of Medicine, University of Fribourg, Fribourg, 1700 Switzerland; 2 Department of Biochemistry, Medical Research Institute, University of Alexandria, Alexandria, Egypt

**Keywords:** Developmental biology, Genetics, Molecular biology, Endocrinology, Molecular medicine

## Abstract

The chromobox homolog 2 (CBX2) was found to be important for human testis development, but its role in the human ovary remains elusive. We conducted a genome-wide analysis based on DNA adenine methyltransferase identification (DamID) and RNA sequencing strategies to investigate *CBX2* in the human granulosa cells. Functional analysis revealed that *CBX2* was upstream of genes contributing to ovarian function like folliculogenesis and steroidogenesis (i.e. *ESR1*, *NRG1*, *AKR1C1*, *PTGER*2, *BMP15*, *BMP*2, *FSHR* and *NTRK1/*2). We identified *CBX2* regulated genes associated with polycystic ovary syndrome (PCOS) such as *TGFβ*, *MAP3K*1*5* and *DKK1*, as well as genes implicated in premature ovarian failure (POF) (i.e. *POF1B*, *BMP15* and *HOXA13*) and the pituitary deficiency (i.e. *LHX4* and *KISS1*). Our study provided an excellent opportunity to identify genes surrounding *CBX2* in the ovary and might contribute to the understanding of ovarian physiopathology causing infertility in women.

## Introduction

The ovarian development depends on a highly orchestrated chain of genetic events involving multiple transcription factors and genetic circuits. Disruption of this orchestrated network can lead to many clinical syndromes, including POF, polycystic ovarian syndrome (PCOS), ovarian hyperstimulation syndrome, ovulation defects, poor ovarian reserve, and ovarian cancer^1^. The genetic regulatory cascade still lacks a master regulator as an equivalent of *SRY* (Sex-Determining Region Y) gene^2^ in the male pathway. Genes such as wingless-type MMTV integration site family, member 4 (*WNT4*)^3^, R-spondin1 (*RSPO1*)^4^ and Forkhead box L2 (*FOXL*2)^5^ are female-specific genes governing the ovarian pathway in coordination with other genes to promote and maintain oocytes health during fetal ovary development^6^. In typical 46, XX female embryonic differentiation, *FOXL2* and the *β-catenin* pathway stimulated by *WNT4* and *RSPO*1, inhibit *SOX9* action, blocking the differentiation of cells into Sertoli cells^7^.

We recently identified *CBX*2 as being upstream of *SRY* and essential for male sex development^8,9^. This homolog of the murine polycomb-like gene *M33* is a highly conserved chromatin modifier^10,11^ and a regulator of homeotic gene expression during early embryogenesis^12^. In humans, two isoforms of *CBX2* have been identified^13^, the long *CBX2* isoform-1 containing a polycomb (Pc) box and the short *CBX2*.*2* isoform lacking the Pc box^13^. Nonetheless, the individual regulation of these different isoforms remains mainly unknown and require further investigation and clarification. A previous study reported that both isoforms can function as repressors of reporter gene activity when bound proximal from a promoter^13^. CBX2.2 does not bind to CBX2.1 and was found to be significantly less active than the long isoform^10,13^. In humans, deficiency in CBX2 represents an autosomal-recessive cause of 46,XY disorders of sex development (DSD)^8^. The 46,XY DSD CBX2.1 deficient patients had normal female internal and external genitalia and ovarian-like tissue at histology^8^. More recently, the description of 46,XX DSD patient with gonadal dysgenesis suggested that CBX2.1 is essential for gonad formation in both sexes. Concerning CBX2.2, 46,XY DSD patients carrying genetic variants of CBX2.2 presented severe testicular dysgenesis phenotype^9^, different from the ovarian‐like gonadal phenotype found in the 46,XY DSD CBX2.1 deficient patient^8^. In mice, while *Sry*-positive *Cbx2* XY^−/−^ animals showed male-to-female sex reversal^14^, knock out *Cbx2* XX^−/−^ animals exhibited gonadal growth retardation and germ cell loss and a high proportion of oocytes with abnormal synapsis and non-homologous interactions which resulted in small ovaries and infertility^14,15^.

To provide further enlightenment about the molecular basis relating *CBX2* to the ovary, we investigated the whole transcriptome associated with *CBX2*.1 and *CBX*2.*2*, which could advance our understanding of the ovarian development, disease and ultimately promote optimal women's health.

## Results

### Gene Ontology (GO) analysis of CBX2.1 and CBX2.2 targets

**Figure 1:**
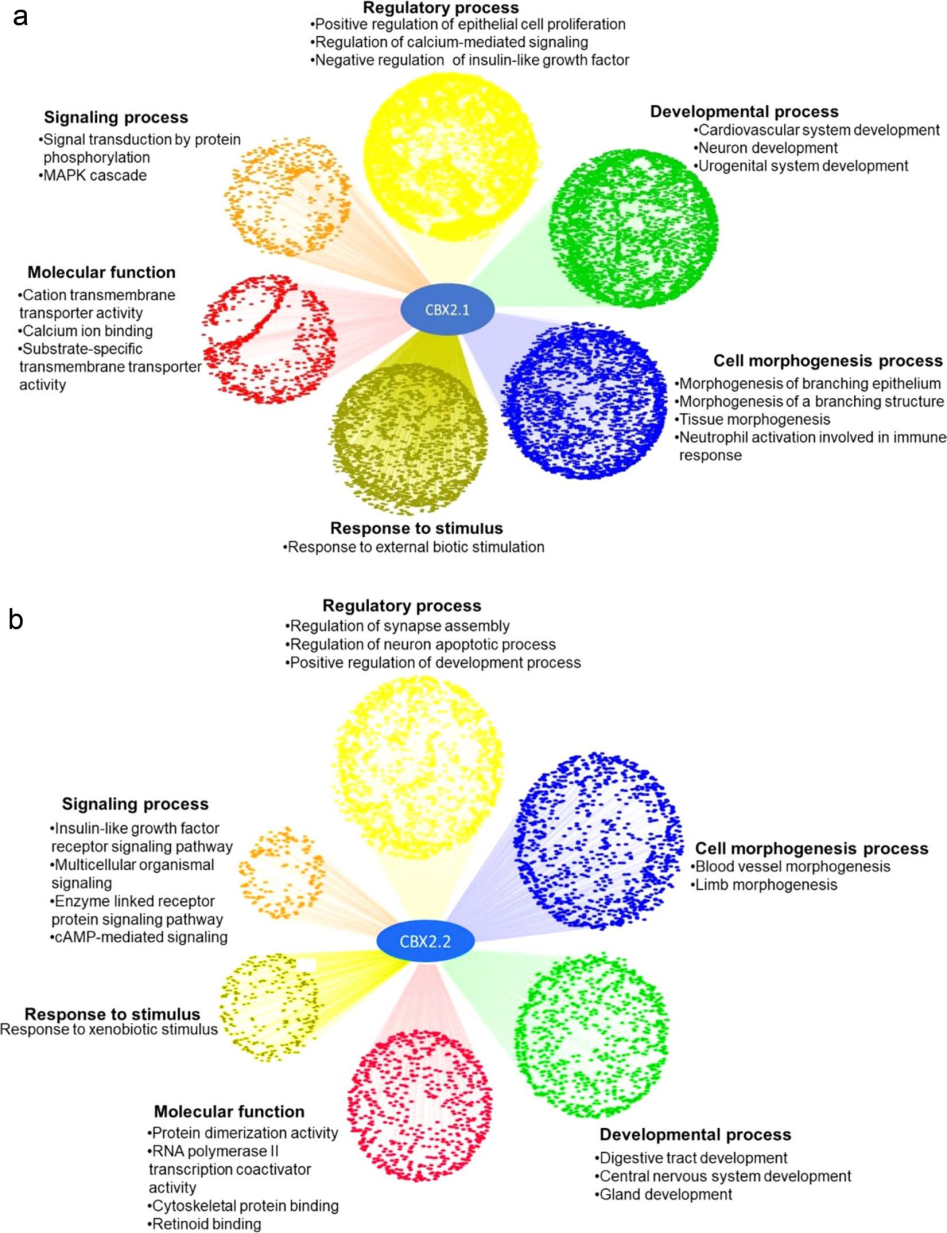
(**a**) Cytoscape representation of GO-enrichment analysis of CBX2.1 targets. Every dot represents a gene related to the enriched GO‐terms. In green are the GO‐terms over-presented in the developmental process. In blue are the GO‐terms involved in morphogenesis process. In red are all GO-terms related to Molecular Function. The orange colour represents the cluster of genes coding for signalling pathways. Some regulatory processes were over-represented by the yellow colour. The genes presented in the mustard colour were over-represented in response to a stimulus. All data is filtered according to p < 0.05. (**b**) Cytoscape representation of GO-enrichment analysis of CBX2.2 targets. The green colour represents the GO‐terms which are involved in morphogenesis and differentiation process. In the blue cluster, we found GO‐terms involved in the developmental process. The red colour indicates genes responsible for Molecular Function. The yellow colour represents the regulatory processes. The orange colour is the cluster, which contains genes coding for signalling processes. The genes present in the mustard colour cluster were over-represented in response to a stimulus. All data is filtered according to p < 0.05.

To gain a functional profile of the high-throughput gene sets obtained from DamID and RNA sequencing, unbiased enrichment analysis was classified into sets of genes with over-represented gene ontology terms. We used ToppCluster^16^ to analyse functional GO-enrichment of *CBX2*.*1* and *CBX*2.*2* downstream genes^16^. Our enrichment analysis indicated multiple genes of *CBX2*.1 and *CBX*2.*2* specifically enriched in generic development, morphogenesis and differentiation of the brain, digestive tube, and glands (Fig. 1a,b). We showed that *CBX2*.1 targets are over-represented for GO-term associated with urogenital system development (Fig. 1a) and that CBX2.1 and CBX2.2 regulate neuronal differentiation by directly interacting with several neuro-associated genes. We identified significant enrichment of genes involved in immune responses through the activation of leukocytes and neutrophils (Fig. 1a). We found a strong enrichment of CBX2.2 related genes involved in the retinoid-binding activity (Fig. 1b) revealed to be crucial during the early female embryonic development^17^. Other *CBX*2.*2* genes were involved in regulatory and signalling processes (Fig. 1b) mediated cyclic adenosine monophosphate (cAMP). This pathway is one of the multiple pathways modulating the ovarian steroidogenesis by increasing the expression of steroidogenic acute regulatory protein 1 activity (StAR)^18^.

### Protein/DNA interaction and transcriptome: Crossover

**Figure 2:**
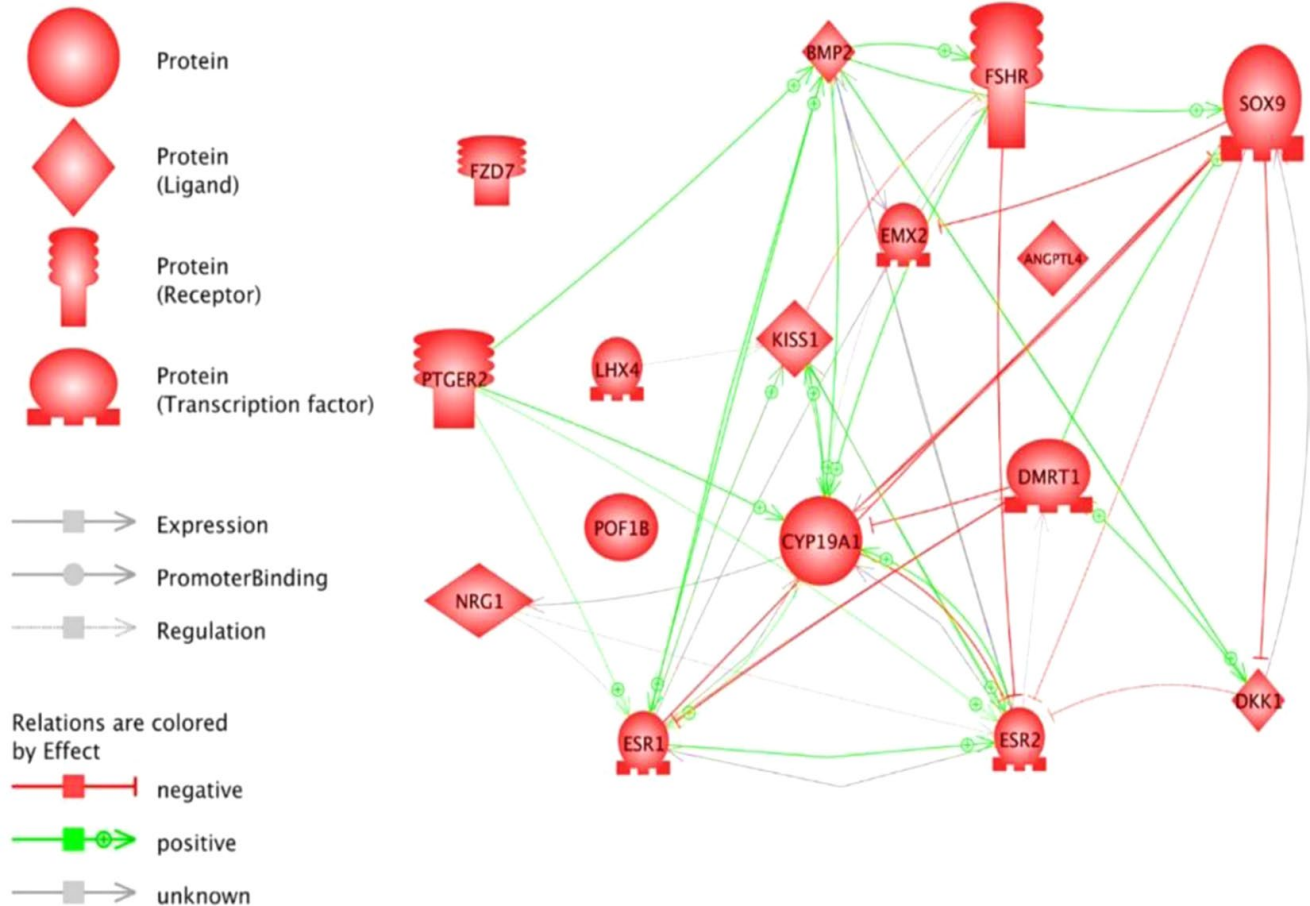
Regulatory network of downstream targets of *CBX2*.*1* in the ovary. We created by the mean of PathwayStudio 11 a network relating the ovarian targets. The genes were found highly interconnected. *CBX2*.*1* targets are *SOX9*, *POF1B*, *DKK1*, *ANGPTL4*, *CYP19A1*, *DMRT1*, *KISS1*, *EMX2*, *ESR2*, *POU4F1*, *FZD7*, *ESR1*, *NRG1*, *BMP2*, *PTGER2* and *FSHR*. The interactions of the genes are represented by diverse arrows. Red arrows (T) indicate negative regulations, the green arrows symbolize positive regulations and grey arrows are undefined effects.

**Figure 3:**
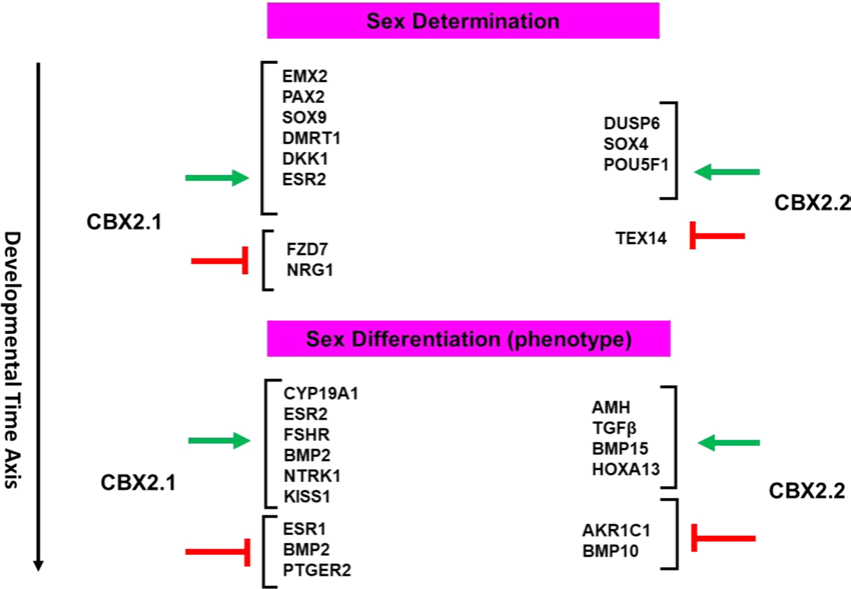
Potential role of CBX2.1 and CBX2.2 in the regulation of sex development. The developmental stages plotted in relation to time are indicated as sex determination. We presented male and female factors involved in gonadal development. Green arrows indicate stimulatory effects of *CBX2*.*1* and *CBX2*.*2* on examined downstream factors; the red arrows (T) indicate inhibitory effects.

We identified common genes regulated by CBX2.1 and CBX2.2 represented in the Venn diagram (Cytoscape 3.7.1)^19^ (Supplementary Fig. 1). For the effects of the two isoforms analyses, we used Fold Change> 2 as the criterion for determining the set of the common genes that exhibit differential expression and *p-*value has been set for all comparisons to be *p* < 0.05. More specifically, the combination of *CBX2*.*1*-DamID targets and* CBX2.1*-RNA-seq related genes showed in total, 53 common genes (Supplementary Fig. 1). About *CBX*2.*2* genes, the intersection between the groups of regulated targets derived from DamID and RNA-seq resulted in 27 up and downregulated common genes in the intersections A, B and D (A∩B∩D) (Supplementary Fig. 2). We defined A as the intersection between the DamID-overexpression of *CBX2*.*1* or *CBX*2.*2* genes and RNA-seq-knock down of *CBX2*.*1* or *CBX2*.*2* genes. The group of genes B is the intersection between the DamID-overexpression of *CBX2*.*1* or *CBX2*.*2* genes and the RNA-seq-overexpression of *CBX2*.*1* or *CBX2*.*2* related genes. The group of genes C resulted in the combination between the RNA-seq-knock down of *CBX2*.*1* or *CBX2*.*2* regulated genes and the RNA-seq-overexpression *CBX2*.*1* or *CBX2*.*2* regulated genes. Group D: is the intersection between the three sets: A, B and C. There were relatively few differentially expressed genes (95 genes) in common between the *CBX2*.*1* and *CBX2*.*2* direct regulated genes obtained from unbiased DamID data (Supplementary Table 1). We recognized 481 overlapped genes acting in diverse pathways between *CBX2*.*1* and *CBX2*.*2* targets resulted from RNA-seq experiments as indicated in Table 2 of the supplementary. This result suggested overlapping pathways of the two isoforms and indicated that *CBX2* isoforms could co-regulate ovary development-specific genes. Thus, we obtained a novel genetic network in which the two isoforms were acting directly or indirectly in the ovary (Fig. 2). The regulated genes have been shown to influence gonad development, apoptosis, proliferation and differentiation processes (Fig. 3). As represented in Table 1, we were able to provide the most up and downregulated genes by CBX2.1 and CBX2.2 which were up to now new in the scene of sex development network.

**Table 1:** Top downstream targets of CBX2.1 and CBX2.2 obtained from DamID and RNA-seq data.

Gene symbol	−log10(p-value)	Fold enrichment	Gene symbol	log2 Ratio	fdr	p-Value
**CBX2.1 DamID**			**CBX2.1 RNA-seq**			
*MYC*	83.89999	8.48561	*RNA5-8S5*	-4.289	8.71E-07	0.001152
*CBX2*	14.52065	7.43178	*RNA5-8S5*	-4.074	8.63E-07	0.001152
*PCDH9*	14.07725	8.75539	*CYP19A1*	2.524	3.04E-07	0.0005027
*MIR1258*	14.07725	8.75539	*ESM1*	1.448	2.83E-07	0.0005027
*GRHL2*	14.07725	8.75539	*PSMA1*	2.349	2.49E-07	0.0005027
*LOC100130331*	14.07725	8.75539	*COL3A1*	-1.482	1.51E-07	0.0004006
*TTC39C*	13.25753	8.31353	*ACTA2*	-1.296	1.50E-07	0.0004006
*YTHDC1*	12.90472	7.73385	*PLIN2*	1.423	1.05E-07	0.0004006
*RCBTB2*	12.64768	8.0819	*ANGPTL4*	3.349	1.81E-24	1.19E-20
*LOC338862*	12.64768	8.0819	*CBX2*	9.212	1.93E-59	2.55E-55
**CBX2**.**2 DamID**			**CBX2**.**2 RNA-seq**			
*LMNB1*	13.63833	8.52171	*MS4A4E*	2.273	1.274E-36	0.00507
*C6orf105*	13.63833	8.52171	*RP11-512M8*.*5*	1.945	1.534E-21	0.003451
*ATP6V1H*	13.63833	8.52171	*ANGPTL4*	1.847	1.629E-20	0.002252
*TRPC7*	13.41263	8.24715	*SHANK1*	5.793	1.629E-20	0.001976
*LOC151162*	13.36989	8.22215	*NYNRIN*	-4.972	1.629E-20	0.001862
*PCDH9*	12.24367	7.86619	*HPCAL4*	5.927	1.543E-19	0.001322
*LPHN2*	12.24367	7.86619	*UQCR11*	2.075	1.543E-19	0.001226
*EPS8L3*	12.24367	7.86619	*DNASE1L2*	6.34	5.678E-19	0.0003375
*LOC151658*	12.24367	7.86619	*C4orf48*	2.908	7.747E-19	0.0001456
*RHO*	12.24367	7.86619	*CBX2*	6.879	5.923E-18	2.66E-17

### Identification of CBX2.1 and CBX2.2 genomic direct and indirect targets in KGN cells

Within 72 hours post-transfection of *CBX2*, pre-granulosa cells or KGN did not exhibit any morphological changes similar to the small and astrocytic shape of the male NT2-D1 cell line morphology^20^ (data not shown). This suggests that there is no link between *CBX2* gene expression and KGN morphological changes. We applied the DamID method that couples whole genome-wide protein-DNA interaction to next‐generation sequencing to gain deeper insights into the function of *CBX2* isoforms in granulosa cells (GC). We identified 524 and 835 enriched binding sequences of *CBX2*.*1* and *CBX2*.*2*, respectively. We expanded *CBX2* transcriptional landscape, by the used of RNA sequencing that identifies, contrary to DamID, also genes that are not necessarily physically bound by CBX2 and can be considered indirect targets. Thus, we found 692 and 668 differently expressed genes, respectively. A larger number of 1167 and 810 genes were significantly up or downregulated by *CBX2*.*1* and *CBX2*.*2* silencing, respectively.

To independently validate the DamID and RNA-seq results, we selected a subset of genes regulated by *CBX2*.*1* and *CBX2*.*2* (as shown in Tables 2 and 3) to evaluate their expression using quantitative real‐time PCR (RT‐qPCR). Genes selection was based on their potential links to sex development, their role in human and animal sexual diseases and their specific expression in tissues involved in sex development (gonads, sex organs, hypothalamus and pituitary).

**Table 2:** DamID selected genes related to CBX2.1 and CBX2.2.

	Target name	Function	Sexual dysfunctions
DamID CBX2.1	*ESR1*	Maintaining the female phenotype of the endocrine somatic cells of the ovary by inhibiting male cell development^128^.	ERKO mice develop testis-like features. Null ERα mutations in human females exhibit profound estrogen resistance and have features analogous to those in the knock out mouse^67^.
	*NRG1*	Induced by luteinizing hormone (LH)/hCG to activate the MAKP3/1 pathway to promote GC differentiation and controls ovulation and luteinization related events^74^.	Not reported
	*BMP2*	BMP2 with FOXL2 ensure expression follistatin in the developing ovary. It amplifies FSH-induced estradiol production in sheep granulosa cell^23^.	In mice, BMP2 null mutation is embryonic lethal and foetuses contain a low number of primordial germ cells leading to POF^129^.
	*PTGER2*	Regulation of ovulation and luteinization^130^.	Mice deficient in Ptger2 have ovulatory defects that are related to an abnormality in cumulus expansion^131^.
	*SOX9*	Stimulates the differentiation of Sertoli cells^132^.	In mice, derepression of Sox9 expression in XX gonads leads to testis development. Human mutation: 46, XY-sex reversal^8^. Duplication: 46,XX DSD.
	*POF1B*	Regulates ovarian function^133^	Assumed to be a causative candidate of POF^134^
	*DKK1*	Repress WNT mediated beta-catenin signalling during the developing testis^135^.	In humans, it is a PCOS risk candidate^136^.
	*FZD7*	WNT signalling regulation^137^	Not reported
DamID CBX2.2	*AMIGO2*	Potential role in lipid metabolism^138^	Not reported
	*TGFα*	Stimulate GC proliferation; inhibit follicle stimulating hormone (FSH) receptor (granulosa cell), and LH receptor (thecal cell) expression; inhibit steroidogenesis^139^.	Not reported
	*TGFb2*	Follicle growth^82^	Polycystic ovary syndrome^82^
	*NTRK2*	Involved in the development and the maturation of the central and peripheral nervous systems^140^.	In the knock out mice reduced the number of secondary follicles and a decrease in granulosa cell proliferation^141^.
	*AKR1C1*	Implicated in the inactivation and formation of male and female sex hormones^142^.	In Akr1c1 deficient mice high progesterone levels and display a delay in parturition of several days^143^.
	*FZD5*	Induce the beta-catenin pathway^144^	Not reported
	*SOX4*	Heart function^145^	Reported in human ovarian cancer^146^
	*RSPO3*	Regulation of Wnt/beta-catenin signalling. It has a possible role in folliculogenesis and development of germ cells of fish^147^.	Not reported

**Table 3:** RNA-seq Genes regulated by CBX2.1 and CBX2.2.

	Genes symbol	Function	Sexual dysfunctions
CBX2.1 Targets	*NTRK1*	Involved in testicular development and spermatogenesis.	Genetic knock out mice resulted in a reduced number of testis cords^148^.
	*ANGPTL4*	Potential role in lipid metabolism^149^	Not reported
	*CYP19A1*	Converts androstenedione to estrone and testosterone to estradiol.	46, XX: virilization of external genitalia^150^
	*DMRT1*	Expressed only in the genital ridge. A dose-dependent effect on postnatal testis development.	XY null mice have normal prenatal testis development, but abnormal postnatal testis differentiation. In human: 46, XY testis maldevelopment; 46, XX primary hypogonadism^151^
	*EMX2*	Homeodomain transcription factor EMX2 is critical for the central nervous system and urogenital development.	Emx2 mutant mice died soon after birth because of the absence of kidneys indicating an essential role in the morphogenesis of the urogenital system^58^.
	*ESR2*	Nuclear receptor transcription factors have a crucial role in reproductive function.	Subfertility and reduced litter sizes and granulosa cell defect^32^.
	*KISS1*	Implicated in the stimulation of gonadotropin-releasing hormone (GnRH)-induced gonadotropin secretion.	KISS1 knock out female mice demonstrated an abnormal reproductive system with abnormal reproductive system phenotype. A clinical case of hypogonadotropic was associated with a loss-of-function of KISS1^48^.
	*BMP2*	BMP2 with FOXL2 ensure expression follistatin in the developing ovary. It amplifies FSH-induced estradiol production in sheep GC.	In mice, *BMP2* null mutation is embryonic lethal and foetuses contain a low number of primordial germ cells leading to POF^129^
	*LHX4*	Acts as a transcriptional regulator that is involved in the control of differentiation and development of the pituitary gland.	In human impaired sexual development, Lhx4−/− double-mutant exhibited a specific abnormal placentas phenotype^152^.
	*POF1B*	Regulates ovarian function	Assumed to be a causative candidate of POF^153^
	*FSHR*	Follicle stimulating hormone receptor	Mutations in the FSHR cause primary ovarian failure in females and impaired spermatogenesis in males^50^.
CBX2.2 Targets	*BMP15*	Stimulation of ovarian granulosa cell growth and proliferation and downregulates FSH receptor expression.	In mice: subfertile, in human: ovarian dysgenesis^154^.
	*TEX14*	Involvement in spermatogenesis and male fertility.	Male mice infertility^96^
	*BMP10*	Inducer of trophoblast differentiation in human.	Not reported
	*MAP3K15*	Involved in the adrenal pathway	Not reported
	*HOXA13*	Required for morphogenesis of terminal gut and urogenital tract, including Müllerian structures.	Mouse: XX null has hypoplasia of the cervix and vagina. 46, XX human mutation: a hand-footgenital syndrome with uterine malformation^89^.

### CBX2.1 downstream targets

**Figure 4:**
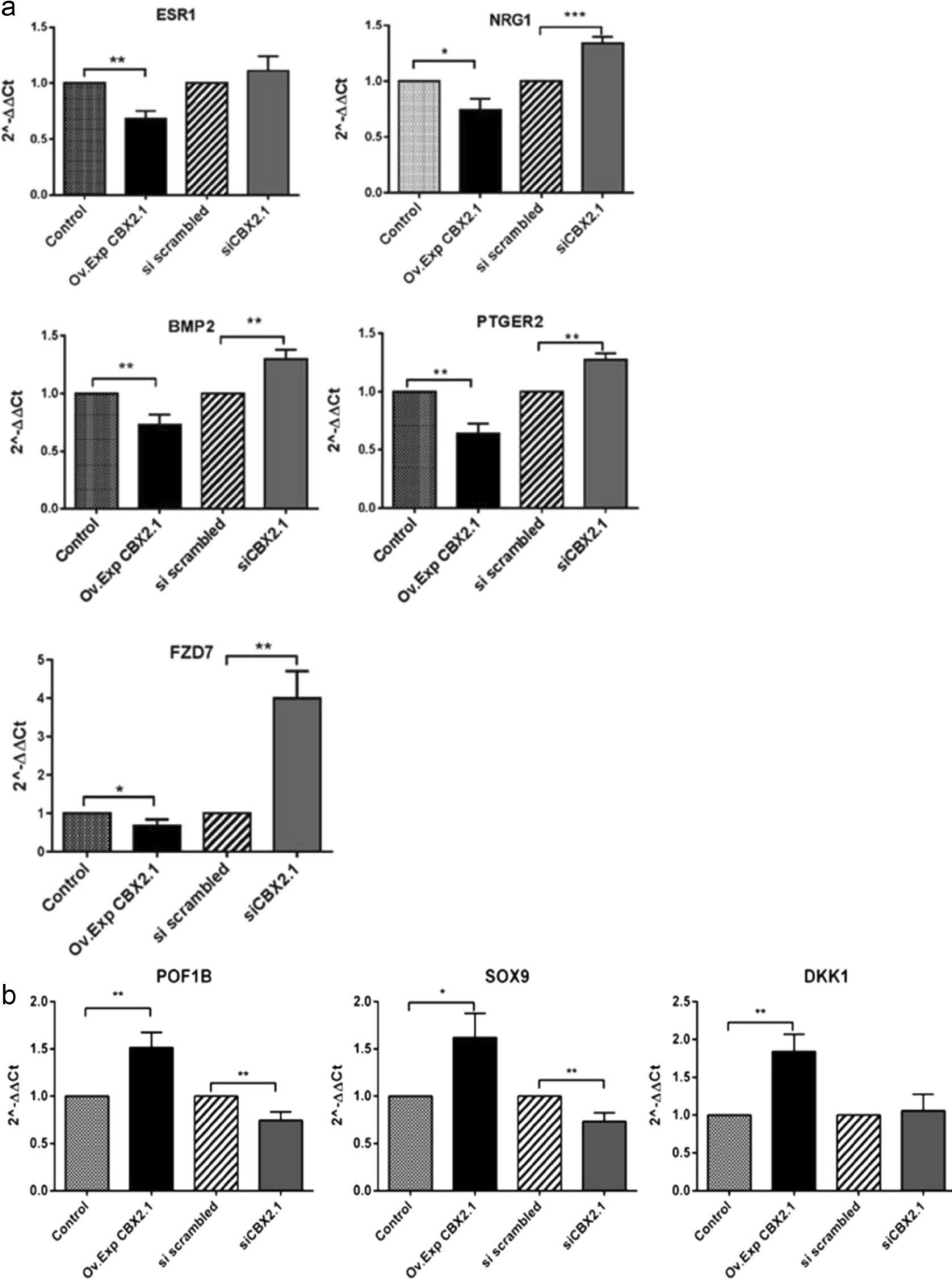
(**a**) RT-qPCR analysis of *CBX2*.*1* downstream genes identified by DamID. Relative expression levels (2^−ΔΔC*t*^) of the genes were determined after normalization to Glyceraldehyde-3-Phosphate Dehydrogenase (*GAPDH*). Following CBX2.1 overexpression (Ov Exp. CBX2.1), *ESR1*, *NRG1*, *BMP2*, *PTGER2*, and *FZD7* were found downregulated by *CBX2*.*1* compared to the control set at 1. After silencing the *CBX2*.*1* (si *CBX2*.*1*), genes were significantly upregulated except for *ESR1* which showed an effect comparable to scrambled sample. All graphs are the average of three independent experiments, error bars represent the standard deviation (SD) from the mean (SEM) and values are expressed as relative to control =1; ***P < 0.001; **P < 0.01 and *P < 0.05. non-significant differences are not indicated. (**b**) Relative expression levels (2^−ΔΔC*t*^) of *CBX2*.*1* related genes. *POF1B*, *DKK1* and *SOX9* were upregulated after *CBX2*.*1* forced expression (Ov. Exp *CBX2*.*1)*. Whereas, when *CBX2*.*1* was silenced (si *CBX2*.*1*), *SOX9* and *POF1B* were significantly downregulated. *DKK1* did not show any expression change towards the siCBX2.1. All graphs are the average of three independent experiments, error bars represent SD from the mean (SEM), and values are expressed as relative to control =1; ***P < 0.001; **P < 0.01 and *P < 0.05. non-significant differences are not indicated.

The genes *ESR1*, *NRG1*, *BMP*2, *PTGER*2, *FZD7*, *POF1B*, *DKK1* and *SOX9* are DamID-CBX2.1 downstream targets. The set of genes, namely *ESR1*, *NRG1*, *BMP*2, *PTGER*2, *FZD7* were found to be negatively regulated by CBX2.1 (0.4-, 0.3-, 0. 35-, 0.3- and 0.44-fold, respectively, compared to the control vector (Fig. 4a). The genes were reported to be implicated in the female sex development and were found to be controlled by the ovarian specific genes *FOXL*2 and *WNT4*^21–26^ recently shown to be downregulated by CBX2.1 isoform^66^. The expression levels of *NRG1*, *BMP*2, *PTGER*2, and *FZD7* but not *ESR1* were significantly increased after *CBX2*.1 knocking down (1.33-, 1.29-, 1.27-, and 4-fold, respectively) (Fig. 4a). CBX2.1 activated *POF1B*, *DKK1* and *SOX9* gene expressions (1.51-, 1.62- and 1.84-fold, respectively) (Fig. 4b). Of particular interest, *SOX9* an essential male-specific gene was demonstrated to be a positive downstream target of *CBX*2.*1*^8,27^ in the testis developmental pathway^28^. Inversely, expression levels of *SOX9* and *POF1B* were shown to be significantly reduced following *CBX*2.*1* silencing (about 0.3- and 0.35-fold, respectively) (Fig. 4b).

**Figure 5:**
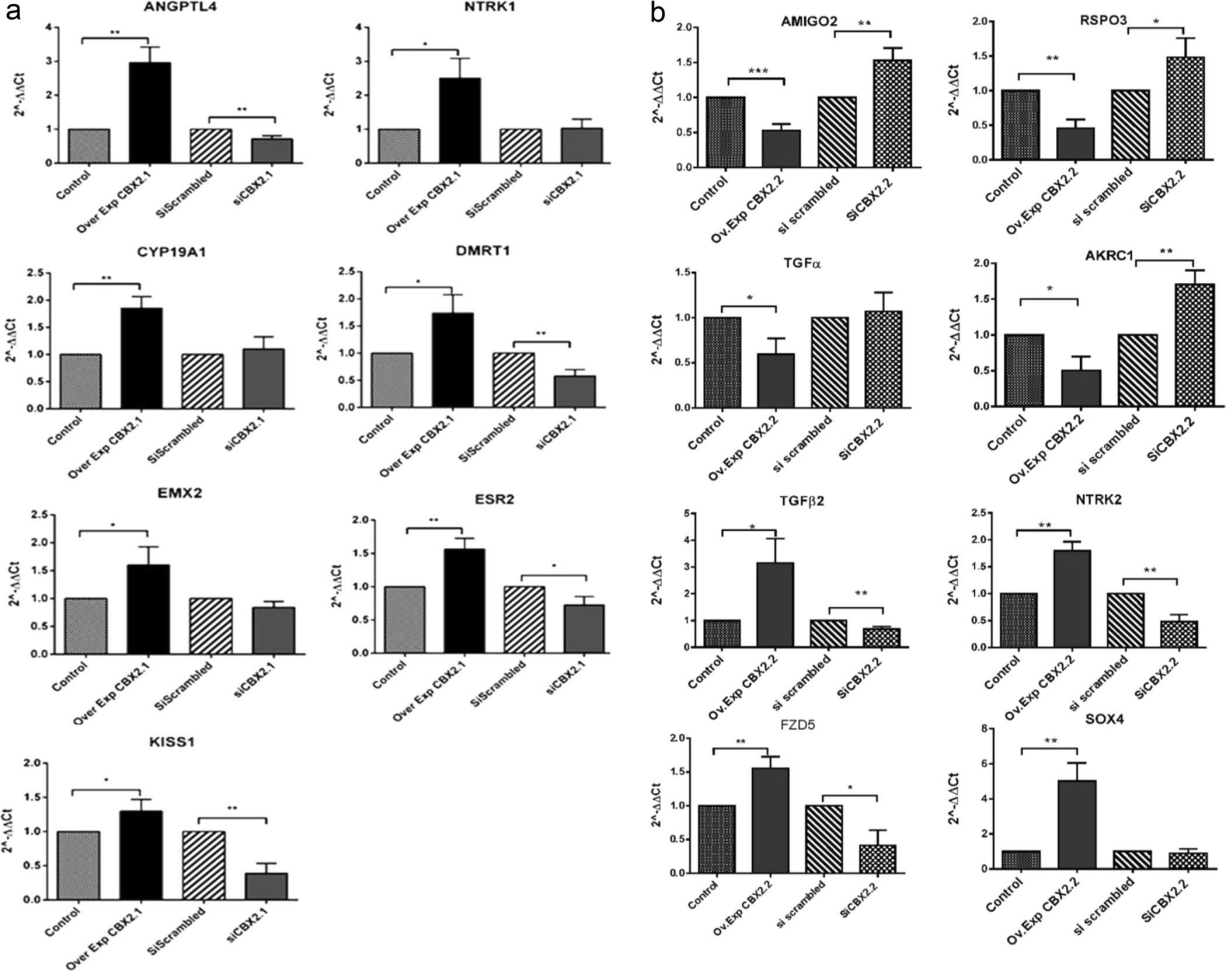
(**a**) Relative expression levels (2^−ΔΔC*t*^) of the RNA-seq of CBX2.1 downstream genes. *ANGPTL4*, *NTRK1*, *CYP19A1*, *DMRT1*, *EMX2*, *ESR2* and *KISS1* were upregulated after *CBX2*.*1* overexpression (Over Exp). *CBX2*.*1* silencing assay (si*CBX2*.*1*) reduced significantly the genes: *ANGPTL4*, *DMRT1*, *ESR2* and *KISS1*. *NTRK1*, *CYP19A1* and *EMX2* gene did not show any expression in response to si*CBX2*.*1*. All graphs are the average of three independent experiments, error bars represent SD from the mean (SEM), and values are expressed as relative to control =1; ***P < 0.001; **P < 0.01; *P < 0.05. non-significant differences are not indicated. (**b**) Effect of *CBX2*.*2* on DamID downstream targets: *AKRC1*, *TGFα*, *AMIGO2* and *RSPO3*. Gene expression levels showed a substantial downregulation after *CBX2*.*2* overexpression (Ov. Exp). The silencing of CBX2.2 (si *CBX2*.*2*) significantly stimulated the expression of *AMIGO2*, *RSPO3* and *AKR1C1* genes compared to scrambled siRNA. *TGBβ2*, *NTRK2*, *FZD5* and *SOX4* genes were significantly upregulated by *CBX2*.*2*. In the si*CBX2*.*2* samples, *NTRK2*, *FZD5* and *TGFβ2* expression levels were found to be negatively regulated. *TGFα* and *SOX4* expressions showed no effect relative to the scrambled sample. All graphs are the average of three independent experiments, error bars represent SD from the mean (SEM), and values are expressed as relative to control =1; ***P < 0.001; **P < 0.01; *P < 0.05. non-significant differences are not indicated.

Among the RNA-seq genes, we found that *NTRK1*, *ANGPTL4*, *CYP19A1*, *DMRT1*, *EMX*2, *ESR2*, *KISS1*, *POF1B* and *FSHR* (follicle-stimulating hormone receptor) were significantly increased following CBX2.1 forced expression (2.5-, 3-, 1.8-, 1.7-, 1.5-, 1.5-, 1.3, 3.6- and 1.7-fold, respectively) (Fig. 5a). To prove a *CBX2-*dependent expression, we tested the gene expression under *CBX2*.*1* silencing. Substantial downregulation affected *ANGPTL4*, *DMRT1*, *ESR2* and *KISS1* genes (0.3-, 0.4-, 0.3-, and 0.58- fold, respectively) (Fig. 5a). However, *CYP19A*1, *EMX*2 and *NTRK1* seemed not to be affected by *CBX2*.*1* knock down, suggesting their regulation by redundant genetic pathways. We found that *CBX*2.*1* overexpression reduced significantly the relative expression of *BMP*2 (0.49-, fold), whereas *LHX4* gene seemed not to be affected (Fig. 5b). As shown in Fig. 5b, *BMP*2 and *LHX4* genes were found to be remarkably upregulated (3- and 1.4-fold, respectively) after *CBX*2.*1* knocking down.

### CBX2.2 downstream targets

**Figure 6:**
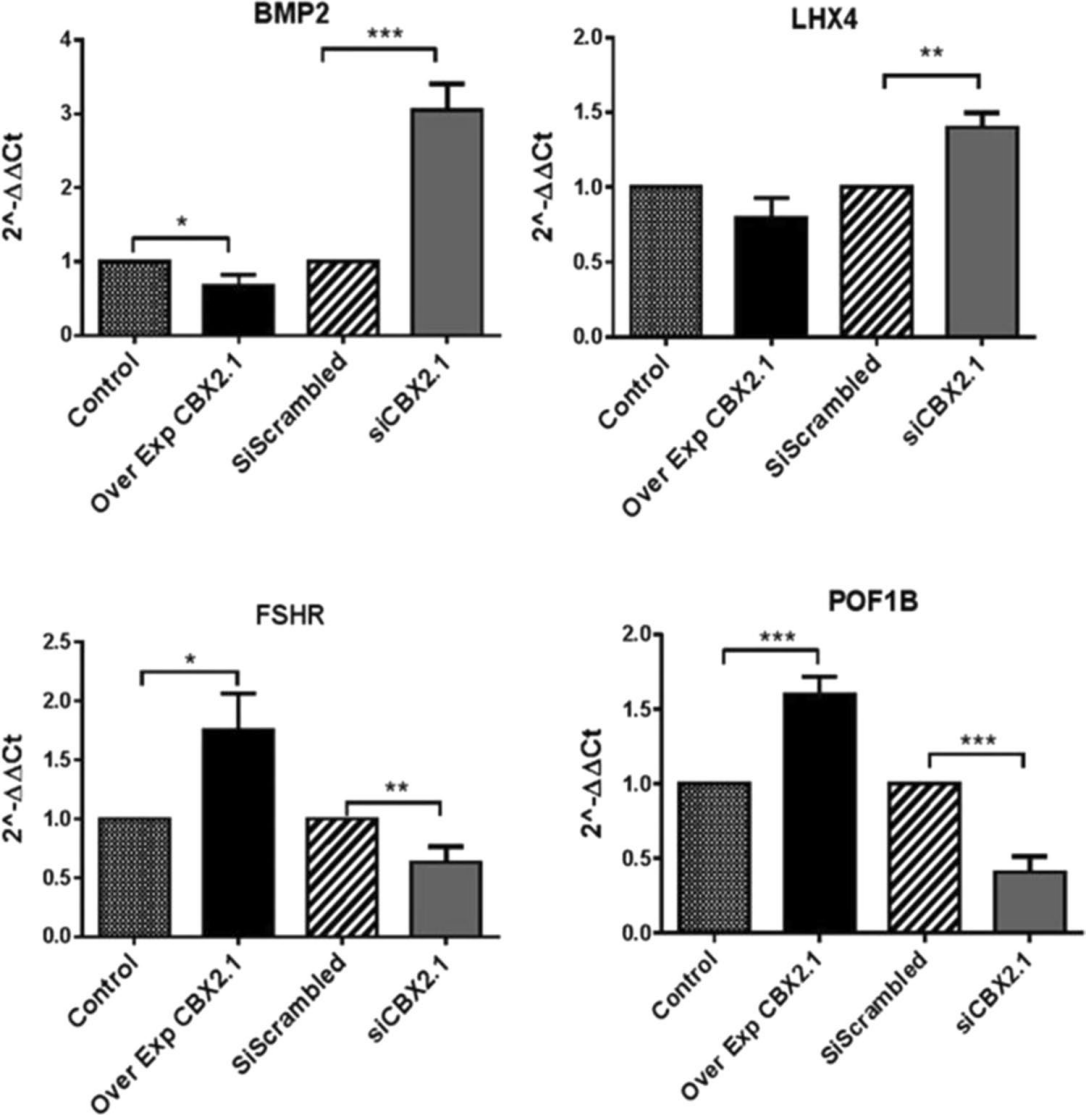
RT-qPCR analysis of RNA-seq downstream genes of *CBX2*.*1*. After CBX2.1 overexpression (Ov. Exp), the relative expression of *BMP2* was downregulated compared to the control (empty vector). *CBX2*.*1* knocking down induced the relative expression of *BMP2* and *LHX4*. However, *POF1B* and *FSHR* were significantly decreased. Forced expression of CBX2.1 does not impact *LHX4*. All graphs are the average of three independent experiments, error bars represent SD from the mean (SEM), and values are expressed as relative to control =1; ***P < 0.001; **P < 0.01; *P < 0.05. non-significant differences are not indicated.

Our result showed that *AKR1C1*, *TGFα*, *AMIGO*2 and *RSPO3* DamID-genes were significantly downregulated by CBX2.2 (0.5-, 0.4-, 0.7- and 0.65-fold, respectively) (Fig. 6). The silencing of the *CBX*2.2 isoform significantly enhanced the expression of *AMIGO2*, *RSPO3* and *AKR1C1* genes **(**2-, 1.5- and 1.7-fold, respectively) compared to the si-scrambled (set at 1). The lack of considerable effects on the expression of *TGFα* after downregulating *CBX*2.*2* is unclear, but it might be attributed to redundant pathways controlling the expression of this gene. On the other hand, another DamID derived-genes *TGBβ2*, *NTRK2*, *FZD5* and *SOX4* were increased after *CBX2*.*2* forced expression (3-, 1.8-, 1.5- and 5-fold, respectively) whereas, under *CBX2*.*2* downregulation *TGFβ2*, *NTRK2*, and *FZD5* expression levels were found to be decreased (0.5-, 0.6-, and 0.3-fold, respectively) (Fig. 6). *SOX4* expression did not exhibit any expression variation after knocking down *CBX2*.*2*.

**Figure 7:**
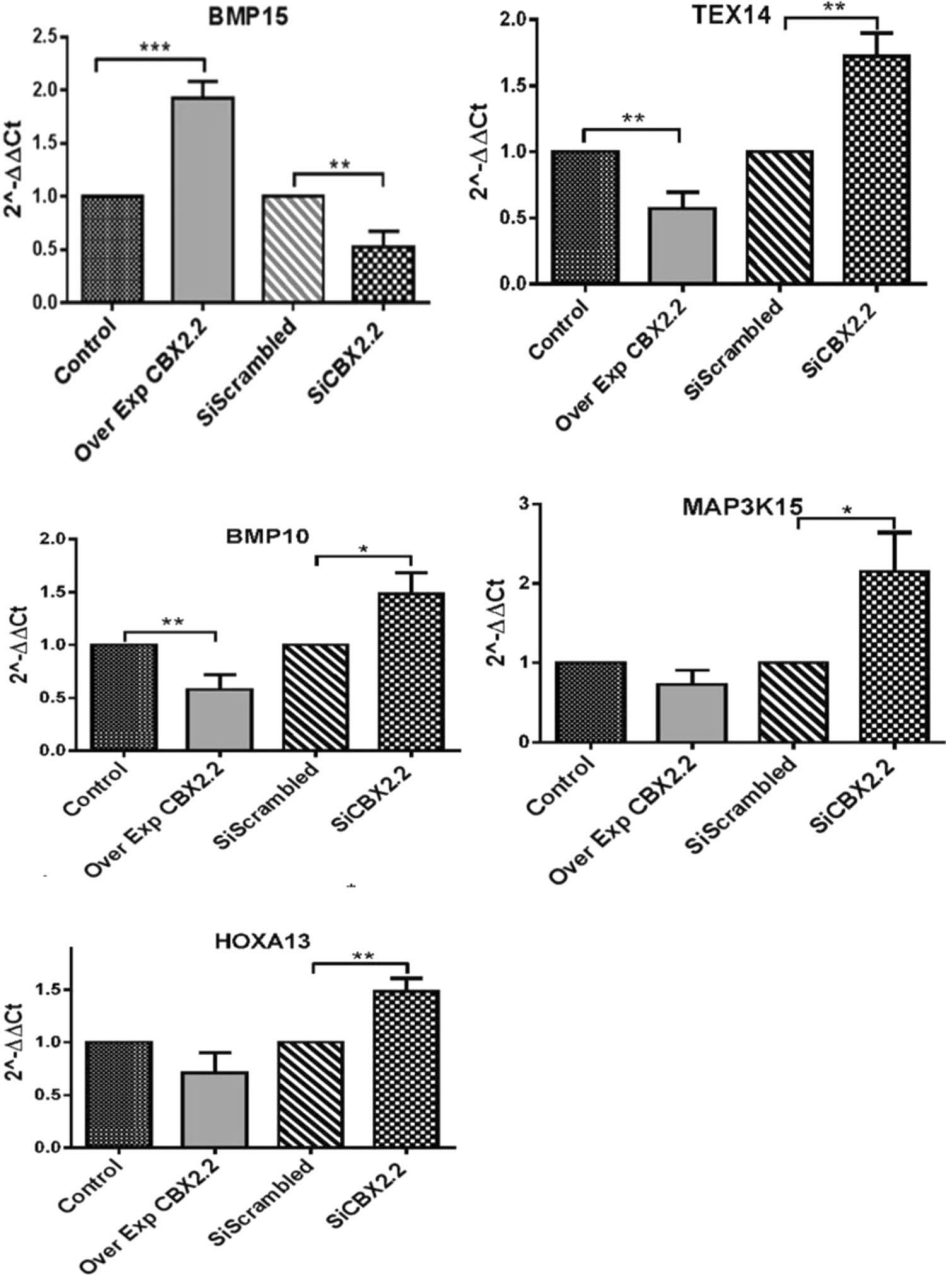
Relative expression levels (2^−ΔΔC*t*^) of the RNA-seq downstream genes of *CBX2*.*2*. *CBX2*.*2* forced expression (Over Exp) positively regulated *BMP15*. Whereas, silencing (si) *CBX2*.*2* (si*CBX2*.*2*) resulted in the downregulation of the same gene. Under overexpression (Over Exp) of *CBX2*.*2*, the genes *TEX14* and *BMP10* were significantly downregulated. Expressions of *MAP3K15* and *HOXA13* were comparable to the control. Silencing *CBX2*.*2* (si*CBX2*.*2*) significantly enhanced the genes *TEX14*, *BMP10*, *MAP3K15* and *HOXA13*. All graphs are the average of three independent experiments, error bars represent SD from the mean (SEM), and values are expressed as relative to control =1; ***P < 0.001; **P < 0.01; *P < 0.05. non-significant differences are not indicated.

The RT-qPCR analysis showed that the ovarian gene *BMP15* was significantly upregulated by 2.2- fold after *CBX2*.*2* forced expression in KGN. Whereas expression of *TEX14* and *BMP10* was significantly reduced after *CBX2*.*2* overexpression (0.33- and 0.40-fold, respectively) (Fig. 7). Silencing of the CBX2 isoform*-*2 decreased the expression of *BMP15* by 0.62-fold. But, it enhanced expression of *TEX14*, *BMP10*, *MAP3K15* and *HOXA13* (1.7-, 1.4-, 2.4- and 3- and 1.6-fold, respectively) (Fig. 7).

It is important to indicate that RT-qPCR did not show that CBX2.1 and CBX2.2 influenced the expression of each other. This agrees with results published by Völkel *et al*., showing that long CBX2.1 isoform interacts with the polycomb repressive complex-1 (PRC1) components. In total contrast, none of the PRC1 components was identified with the CBX2.2 short isoform^13^. According to the same authors, CBX2.2 forms homopolymers in a PRC1-independent way. Unlike CBX2.1, CBX2.2 lacks the Pc domain, essential for the interaction with the PRC1 partners^13^.

## Discussion

In this work, we showed the gene expression landscape of *CBX2* isoforms in the ovary based on data-driven from profiling genes and transcriptome data. Unbiased GO analysis data obtained from the whole genome protein/DNA interaction and RNA-seq methods revealed a greatly expanded “atlas” of *CBX2* new targets implicated in several developmental and functional pathways in KGN. Of particular interest, we demonstrated that *CBX2*.*1* targets are over-represented for GO-term associated with urogenital system development, thereby supporting the involvement of *CBX2*.*1* in human sex development as has been reported by Biason-Lauber and co-workers^8,27^. Several genes with diverse functions related to folliculogenesis, steroidogenesis and ovarian disease like PCOS, POF were found to be regulated directly and indirectly by *CBX2* isoforms. Multiple categories of *CBX2*.*1* and *CBX2*.*2* related genes are implicated in generic development, morphogenesis and differentiation events. Our findings are in harmony with recent data revealing the implication of murine *Cbx2* gene in the central nervous system development in mice^29^. We showed significant enrichment of genes involved in immune responses, supporting the results of Katoh-Fukui in *Cbx2/M33* knock out mice with immunological deficits due to spleen development abnormalities^14^. Yet, no immunological deficit is found to be influenced by CBX2 mutations in human patients^8^. This could be explained by the difference between human and mouse phenotypes and the presence of alternative pathways of *CBX2* gene in humans. Among *CBX2*.*1* and *CBX2*.*2*-regulated genes were factors involved in the regulation of insulin-like growth factor (*IGF*) receptors, which were reported to be required for sex determination in mice^30^. In the double knock out insulin-*Igf1* receptor null embryos, a delay in ovarian differentiation has been observed, suggesting that in mouse gonads lacking insulin/*Igf* signalling remain undifferentiated with no clear pathway decision of either testicular or ovarian pathways for several days^31^.

*CBX2*.*1* related genes were found to be highly interconnected in ovarian developmental processes and supported an active contribution of *CBX2*.*1* in ovarian function and maintenance. Genes regulated by CBX2.1 like *CYP19A1*, *KISS1*, and *ESR2* were found to work together to determine and maintain the ovary phenotype^32–35^. Unlike *CBX2*.*1*, the *CBX2*.*2* network appeared to be much more limited, most likely due to the novelty of CBX2.2's functions and the lack of animal model studies. Taken together, the network-based transcriptome and profiling data offered a solid starting point for the elucidation of detailed connections of *CBX2* isoforms genes in the human ovarian pathway.

A new single-cell RNA-seq analysis using human fetal gonad cells and their neighbouring somatic cells from 15 embryos between 4 and 19 weeks post-fertilization (GEO accession GSE86146)^36^, demonstrated that the *CBX2* transcript is highly expressed in both sexes. The *CBX2* expression level was remarkably higher in the female than in the male embryo^37^. Additionally, human follicular transcriptome data (GSE107746) obtained from various developmental stages of oocytes (primordial, primary, secondary, antral and preovulatory) and the corresponding GC published by Zhang *et al*., showed that *CBX2* expression was consistently high at all stages of follicular development compared with GC^38^. In mice, a study revealed a spectrum of meiotic abnormalities in *Cbx2* deficient oocytes at the diplotene stage with abnormal synapsis and non-homologous chromosome interactions in *Cbx2* (XX^−/−^) mutant oocytes^15^. This phenotype observed in fetal oocytes lacking *Cbx2* might suggest that *Cbx2* is expressed in follicles and have a functional role for chromatin remodelling required for the establishment and repair of homologous chromosome pairing.

The gene Cytochrome P450 family 19 subfamily A member 1 (*CYP19A1*), estrogen receptor 2 (*ESR2)*, (Kisspeptin) *KISS1* and *FSHR* were found to be the upregulated by *CBX2*.*1*. The *CYP19A1* or aromatase A gene is responsible for the aromatization of androgens into estrogens in many tissues in female and male^39^. A previous animal study showed that *Nr5a1*, also called steroidogenic factor-1 (*Sf-1*), depletion leads to reduced *Cyp19a1* expression and low estradiol levels resulting in testis differentiation in the XX gonad^40^. The gene *ESR2* was associated with follicular growth^41^. Mutations of *ESR2* were found to be responsible for 46,XY and 46,XX DSD, both with gonadal dysgenesis^42^. The genes *KISS1* and *FSHR* were related to ovarian diseases like POF and PCOS^43,44^. Recent studies have shown that kisspeptin-1 and its receptor are expressed in the mammalian ovary and are critical for initiating puberty and regulating ovulation in sexually mature females *via* the central control of the hypothalamic-pituitary-gonadal axis^45^. Data gathered recently suggested a putative role of kisspeptin signalling in follicular development, oocyte maturation, steroidogenesis, and ovulation^46^. Additionally, loss-of-function of *KISS1* is associated with hypogonadotropic hypogonadism leading to reproductive function failure and female infertility^47,48^. As for *FSHR*, it plays a major role in the development of follicles and steroidogenesis in the ovary^49^ and a loss-of-function of *FSHR* causes ovarian dysgenesis^50^.

We identified upregulated factors such as *SRY*-box 9 (*SOX9)* and doublesex and mab-3 related transcription factor 1 (*DMRT1)*^8,27,51,52^ as CBX2.1 downstream masculinizing factors. Heterozygous loss-of-function mutations in human *SOX9* cause sex development disorder in XY males^53,54^ while gain-of-function mutations, such as gene duplication, can lead to XX female DSD^55^. According to Ledig and co-workers, a partial deletion of *DMRT1* causes 46,XY ovotesticular sexual disorder^56^. The ovotestis formation is caused by the disturbed action of *DMRT1* in germ cells as well as in Sertoli cells, causing female reprogramming of the testis^56^.

Other genes like empty spiracles homeobox 2 also known as *EMX2*, Dickkopf-related protein 1 (*DKK1)* and neurotrophic receptor tyrosine kinase 1 *(NTRK1)* are gonadal dual-functional factors upregulated by *CBX2*.*1* in ovarian GC. It has been reported that *EMX2* is indispensable for the formation of the embryonic structures Müllerian and Wolffian ducts in the female and male embryo^57,58^. A nonsense mutation of *EMX2* resulted in uterus didelphysis in Chinese women with incomplete Müllerian fusion^59^. In agreement with our data, a murine study showed that *Emx2* is downregulated in *Cbx2*-deficient gonads^14^. In women, genetic variation in *DKK1* may result in hyperandrogenism and metabolic dysfunction of PCOS^60^. Other data suggested that mice *Dkk1* plays a backup or fail-safe role in preventing Wnt signalling which is in harmony with the possible antineoplasic role of CBX2^61^. We also found that CBX2.1 stimulated *NTRK1* expression, which has been reported to be involved in the assembly of primordial follicles^62^ to facilitate the progression of follicular development within the ovary^62^. Together, our data indicate that CBX2.1 might be required within ovarian cells for follicular fate regulation which agrees with previous findings showing *Cbx2* mutant female mice with small ovaries and significant germ cell loss^11^.

We found angiopoietin-like-4 (*ANGPTL4)*, a factor yet novel to the scene of sex development, that we found it upregulated by CBX2.1 in GC. Importantly, murine *Angptl4* has been found to play a role in lipid metabolism, which can provide cellular energy and mobilize substrate for progesterone synthesis in breeding females^63^. Together, we suggested a putative correlation between *CBX2*.*1* and *ANGPTL4* to maintain hormone metabolism in the ovary. *ANGPTL4* has been also reported to be an apoptosis survival factor capable of preventing metastasis by inhibiting vascular growth and protecting from tumour cell invasion^64^. The explanation of the role of *ANGPTL4* in ovarian physiopathology, if any, seems to be more challenging.

Some of the most important factors negatively regulated by *CBX2*.*1* are estrogen receptor 1 (*ESR1)*, prostaglandin E receptor 2 (*PTGER2)* and bone morphogenetic protein 2 *(BMP2)*. The genes seem to be interconnected with *FOXL2*^65^. CBX2.1 was demonstrated to downregulate the female determining factor *FOXL2* in testis and ovary gonads^27,66^. *ESR1* was reported to cooperate with *FOXL2* to restrain *SOX9* in the ovary^65^. In women, *ESR1* deficiency was associated with clinical features of estrogen resistance, including primary amenorrhea, the absence of breast development, a small uterus and enlarged multicystic ovaries^67,68^. In humans, *PTGER2* and *BMP2* gene are prerequisite for ovulation and are activated by female factor *FOXL2*^21,69^. The repressive effect of *CBX2*.*1* on these ovarian factors together with the stimulation of male-typical factors, such as *SOX9* and *DMRT1*, indicate a sort of dual-function of *CBX2*.*1* in the development of the human gonads. It seems not to be an isolated example. Other genes like the *WNT4*, *ESR2* and *SF-1* have a necessary role for ovarian and testicular development function^70–72^ as demonstrated by the fact that genetic variants in these genes cause gonadal dysgenesis in 46,XY individuals^70,73,74^, with ovarian failure in women and ovotesticular DSD^42,70,74^. Some authors suggested that sex determination is sensitive to gonad genes dosage at multiple steps in the gonads pathway^75^, which might be the case of the *CBX2* gene. Recent preliminary reports of *CBX2*.*1* genetic variations in 46, XX individuals with gonadal abnormalities lend further weight to an essential role of *CBX2* in human ovarian and testicular development^76^.

There is very little information available about the role of the second isoform *CBX2*.*2* in any process in women. Recently, Sproll *et al*. showed the existence of two *CBX2*.*2* genetic variants that fail to regulate the expression of genes essential for sexual development, leading to a severe 46,XY DSD defects^9^.

Among the factors upregulated by CBX2.2, were the bone morphogenetic protein 15 *(BMP15)* and the transforming growth factor-beta 2 *(TGF-β2)* genes. Previous studies showed that *BMP15* affected the production of estradiol and progesterone^77–79^. Studies in animal have shown that the activation of the primordial follicles is mediated by *bmp15*^80^. In humans, the defected *BMP15* in patients was found to cause ovarian failure^81^. Mounting evidence supported the implication of *TGF-β2* in female reproduction and development^82^. *TGF- β* superfamily members may play different roles in the development of follicles across the species^80^. *Tgf-β2* knock out mice study showed multiple developmental defects, including cardiopulmonary, skeletal, ocular, and urogenital system defects^83^. In women, dysregulation of this transforming growth factor circuitry was associated with PCOS^84,85^ and fertility problems^86^. These findings may point to potential roles of *CBX2*.*2* in regulating transforming growth factors networks reportedly found crucial during follicular development^38,87,88^.

In the present study, CBX2.2 upregulated dual-functional factors in gonads like the early expressing gonadal factors, *Homeobox* protein Hox-A13 (*HOXA13)* and *SRY*-box 4 (*SOX4)*. In humans, *HOXA13* mutations were found to affect uterine development^89,90^ and produced hand-foot-genital syndrome in females^91^ with a decrease in androgen expression in males^90^. These data imply that *CBX2*.*2* might play a role in the normal expression of *HOXA13* in the early developing ovary, mirroring the situation in the human testis^92^. In our hands, the group C SOX transcription factor *SOX4* is downstream of *CBX2*.*2*. In mice, *Sox4* deficiency results in abnormal gonads of both ovaries and testes^93^. A recent animal study revealed that *Sox4* was among *Foxl2* positively regulated genes in the mice ovary^94,95^ and showed to repress transcription of *Sox9* in fetal gonads, raising the possibility that *SOX4* may function as a new feminizing C SOX factor in the regulation of the ovarian determination.

The testis expressed-14 gene *(TEX14)* is a masculinizing factor downregulated by CBX2.2. It has been reported to be required for intercellular bridges in vertebrate germ cells^96^. In females, these embryonic intercellular bridges have been proposed to have a role in the development of the primordial germ cells^96^ and *Tex14*^−/−^ ovaries have fewer oocytes relative to control ovaries in mice^97^. The human fetal ovary expresses *TEX14* after the 12^th^ week of gestation, suggesting that the growth of oogonia may be induced by cellular precursor transport from neighbouring oogonia *via* the *TEX14* channels^98^.

CBX2.2 negatively regulated dual-functional factors as mitogen-activated protein kinase kinase kinase 15 ***(****MAP3K15)* and Aldo-keto reductase family 1 member C1 (*AKR1C1)*. The two genes appear to serve as markers involved in steroidogenesis^99,100^. Reduced levels of activated mitogen-activated protein kinase (*MAPK*) contribute to excessive ovarian androgen production in women with PCOS^101^. Besides, the change of expression patterns of *MAPKs* in rat ovaries was significantly higher during the secondary and antral follicle stages than those in the primordial follicles, primary follicles and corpora lutea indicating their possible involvement during follicular growth and development^102,103^. We assume that *CBX2*.*2* could be implicated in the optimal control of the *MAPKs* signalling pathway in GC during differentiation and proliferation processes. We studied one of the Aldo-keto reductase steroidogenic enzymes, which is the *AKR1C1*. Recently collected data showed that it is expressed in adrenal tissue and may be involved in the fine regulation of androgen and androgen receptors (AR) availability in adipose tissue in men and women^104,105^. Aldo-keto reductases (*AKR1C1-C4*), 5α-reductases and retinol dehydrogenase were found to be expressed in the ovary, indicating that the human ovary might produce dihydrotestosterone *via* the alternative steroid backdoor pathway^106^. This pathway seems to be considerably enhanced in the polycystic ovary syndrome^104^. Little is known about the direct involvement of AR actions in the female. Nonetheless, previous results based on global *AR* knock out female mice^105,107^ demonstrated that they are subfertile, have defective folliculogenesis and ultimately develop POF. In the human ovary, androgen precursors are crucial for estrogen synthesis and hyperandrogenism in pathologies such as the polycystic ovary syndrome^108^. Taken together, our study provides a piece of indirect evidence about the role of *CBX2*.*2* in the regulation of androgen receptor in the ovary that could be through the control of *AKR1C1* expression. However, further studies are paramount to show how these new targets fit into the expanding *CBX2*.*2*-regulated network and how *CBX2*.*2* activation and suppression can impact our understanding of ovarian functionality in humans.

We concentrated our study on the developmental side of *CBX2* since variants of *CBX2* in human leads to developmental defects like gonadal dysgenesis in women and men^8^, Although yet no defect in *CBX2* is known in later stages we suggested that *CBX2* and some of its targets might be involved in adult ovarian dysfunction such as PCOS and POF. To lend more weight to our hypotheses, we compared our selected *CBX2*.*1* and *CBX2*.*2* targets with the existing RNA-seq datasets of female embryonic and mature gonad cells^37^.We found that CBX2 is greatly and specifically expressed during all stages of female fetal gonadal cells (FFGC) including mitotic, retinoid acid (RA) responsive, meiotic, oogenesis, endothelial, early granulosa, mural granulosa and late granulosa phases^38^. Substantially, some of the major *CBX2*.*1* and *CBX2*.*2* downstream targets like *SOX4*, *ANGPTL4*, *AKR1C1*, *BMP2*, *EMX2*, *DMRT1*, *CYP19A1*, *FZD5* and *TEX14* showed also high and specific expression patterns in the same fetal stages. In our study, the *ANGPTL4*, *DMRT1*, *EMX2*, *CYP19A1* genes were found significantly activated by CBX2.1. Using the same available public dataset, *ANGPTL4* and *DMRT1* were abundantly expressed during the FFGC and particularly in the mitotic, RA-responsive and meiotic stages. New findings indicated that in mice the lack of *Dmrt1* in the fetal ovary resulted in the formation of many fewer primordial follicles in the juvenile ovary^109^. *EMX2* and *CYP19A1* showed high expression in the early, mural and late granulosa stages. To the best of our knowledge, *Emx2* is one of the genes which was found to be necessary for the survival of the female and male gonads in mice. Nonetheless, the gene was not reported to play a crucial role at various stages during oocyte development^110^. We showed that CBX2.1 downregulated *BMP2* ovarian marker. According to the transcriptome data of Zhang and co-workers^38^, this gene was found highly expressed during the meiotic, oogenesis, early granulosa, mural granulosa and late granulosa stages. This is not surprising given the well-proven expression of *BMP2* in GC and germ cells^111^. Consistent with this, *BMP2* was reported to be important for the follicular development^111^ and a predictor marker of embryo quality^112^. We demonstrated that genes like *SOX4* and *FZD5* are positively regulated by CBX2.2 and seem to have consistent high expressions during all stages of the FFGC. Noteworthy, a recent mice study indicated that *Sox4* plays an important role in mouse gonad development by promoting gonad germ cell differentiation^93^. The *AKR1C1* and *TEX14* are CBX2.2 downregulated genes and exhibited high expression profiles during all ovarian fetal cells. In the first place, the results indicated that these factors are most likely node genes that establish a robust regulatory network governed by *CBX2* gene in the fetal ovary, which may be involved in the control of these genes pathways during fetal germ cells and their neighbouring GC development^38^. Overall, this data could offer a solid reference dataset for the implication of the *CBX2* and the candidate genes in the regulation of follicular development and could be valuable factors that are important in several stages of ovarian life, spanning from development, maintenance, reproductive potential and function.

A limitation of our study is that we did not use primary cultured human GC, but a KGN tumour cell line. Primary GCs can be isolated from follicular fluid during oocyte retrieval procedures. This retrieval takes place after ovulation when GCs undergo a process of luteinization which involves structural and genomic changes that lead to the terminal differentiation of follicular cells with increased progesterone production^113^. Luteinized GCs stop their proliferation, making the long-term cultivation of GCs primary cells extremely challenging^114^. Furthermore, the differentiation of GC into luteinized cells has effects on intracellular signalling and cell cycle regulation, rendering these cells not suitable to represent developing GCs. The use of the animal models as an alternative remains problematic^37^ as many genes are expressed differently and preferentially in human and mice. Also, Homo sapiens seem to be the only mammalian species to have two CBX2 isoforms. We therefore resorted to the use of cell lines. The KGN cell line is very well characterized^66,115–119^ and represents the most suitable alternative *in vitro* model and the closest to human ovarian cells to ascertain the role of *CBX2*.*1* and *CBX2*.*2* in human ovary cells.

In all, the combination of different hypothesis-generating NGS-approaches allowed us to shed light on the transcriptionally CBX2-dependent landscape in the ovarian pathway. Certainly, new knowledge in *CBX2* network using novel advanced technologies for detecting and sequencing exomes are essential to prove the exact role of the new putative CBX2 regulated genes in the developing ovary network. It would be an important investigation, not only to advance our understanding regarding gonadal development but also to expand our ability to diagnose, counsel and properly accompany patients affected by ovarian dysfunctions like infertility and cancer.

## Methods

### Cell culture

The human ovarian granulosa-like tumour commercially available cell line or KGN^116^ has been provided by RIKEN BRC. It has been established from a tumour specimen enucleated from a 63-year-old woman who was diagnosed with a local recurrence of granulosa cell carcinoma after menopause. A portion of the granulosa tumour tissue obtained was used as the source of the cell culture^116^. We maintained KGN cells in Dulbecco's essential medium/Ham’s F12 medium supplemented with 10% fetal calf serum, 5% Penicillin/Streptomycin at 37 °C in a 5% CO_2_ as described in Nishi *et al*. protocol^116^. Cells were transfected by 2 *µ*g of plasmids encoding for CBX2.1 (SC303599, OriGene Rockville, Maryland, USA), C-Myc-CBX2.2 (RC216313 OriGene Rockville, Maryland, USA) and pCMV6-empty plasmid was used as a control vector (PS100010 OriGene Rockville, Maryland, USA). We transfected cells with Metafectene (Biontex Laboratories, Munich, Germany) with ratio 1:4 (transfection reagent: DNA). siRNA duplexes (purchased from Microsynth) were introduced into cells using Lipofectamine RNAiMAX (Invitrogen) in two consecutive rounds at a final concentration of 40 nM. Experiments were typically performed 48 hours after the first transfection. siRNA duplex for CBX2 isoforms silencing was designed to alter specifically *CBX2*.*1* and *CBX2*.*2* so that siCBX2.1-oligos are not targeting *CBX2*.*2* and vice-versa (si-oligo sequences are available upon request).

### DamID

In principle, the DamID technique is based on *in vivo* expression of a chromatin protein of interest fused to DNA adenine methyltransferase (Dam)^120^. CBX2-Dam identification was achieved as previously described by Eid *et al*. (2015). Briefly, human *CBX2*.*1* and *CBX2*.*2* cDNA (OriGene, Rockville, Maryland, USA) were amplified and cloned then recombined into the destination vector to generate Dam-CBX2 construct. DamID was performed using a lentiviral transduction protocol^120^. Genomic DNA was isolated and used as templates to amplify methylated genomic fragments. DNA libraries were then prepared using the TruSeq DNA LT Sample Prep Kit (Illumina) and the libraries were sequenced on a HiSeq. 2000 sequencer (Illumina).

### Illumina RNA sequencing

RNA sequencing provides far higher coverage and greater resolution of the dynamic nature of the transcriptome^121^. Total RNA was isolated and physical integrity was examined on Agilent Bioanalyser 2100 and Agilent 2200 TapeStation. Three replicates for each sample were performed for transcriptome analysis. RNA library preparation for sequencing was achieved according to the protocols of the functional genomics centre of Zurich (Switzerland)^122^. Typical cut-offs for candidate selection are Log2Ratio>1 (2x Fold) or Log2Ratio <−1 (2x Fold in the other direction) and an FDR (false discovery rate) of 0,05. The resulting *p*-values were FDR corrected using the Benjamini-Hochberg method. Genes with FDR values equal or smaller than ≤0,05 were considered differentially expressed.

### GO enrichment analysis

GO‐terms with *p*‐values ≤ 0.05 and more than five target genes associated with the corresponding GO‐term were defined as significant. *CBX2*.*1* and *CBX2*.*2* target genes were clustered depending on GO‐terms and visualized using a spring-embed layout with Cytoscape v3. 3.0^123^. We used ToppCluster^124^ to explore the functional significance of the binding patterns of *CBX2*.*1* and *CBX2*.*2*. GO‐enrichment permits to analyse functional features of gene sets, clustering them by their involvement in pathways related to Molecular Function, Biological Process and/or Cellular Component. GO-terms were considered as significantly enriched when value equal to or smaller than ≤0,05. Downstream genes were clustered according to their GO-terms. PathwayStudio 11 (Elsevier) is an exhaustive resource of easily searchable data from biology articles describing interactions between molecules, cell processes and diseases^125^. The platform allowed us to analyse the connection of *CBX2* related genes in the human ovary genetic network.

### Quantitative RT-PCR

In this study, we performed the RT-PCR as a technical validation approach. Gene expressions were evaluated using KAPA SYBR FAST qPCR Kit (KAPA BIOSYSTEMS). All samples were run in triplicates and the normalized relative expression values (2^−ΔΔC*t*^)^126^ of multiple independent experiments were plotted against the control vector set at 1. Statistical analyses were conducted using GraphPad Prism version 6.07 (Software, La Jolla, California, USA) and data sets were analysed for statistically significant differences using unpaired Student's *t*-test^127^ with confidence intervals set at 95%. Our gene-specific primer sequences and RT-qPCR conditions for *CBX2* isoforms and selected targets amplification are available upon request.

## Supplementary Information

Supplementary Info
