# Supplementary material for: The transcriptional regulator CBX2 and ovarian function: A whole genome and whole transcriptome approach
Source: Sci Rep. 2019 Nov 19;9:17033. doi: 10.1038/s41598-019-53370-4 (PMC6864077; doi:10.1038/s41598-019-53370-4)
Supplement: Supplementary file 1 — Supplementary Info [file 41598_2019_53370_MOESM1_ESM.pdf]

The transcriptional regulator CBX2 and ovarian function: A whole genome and whole transcriptome approach.

Leila Bouazzi[1], Patrick Sproll*[1], Wassim Eid*[2], Anna Biason-Lauber[1]

Supplementary information:

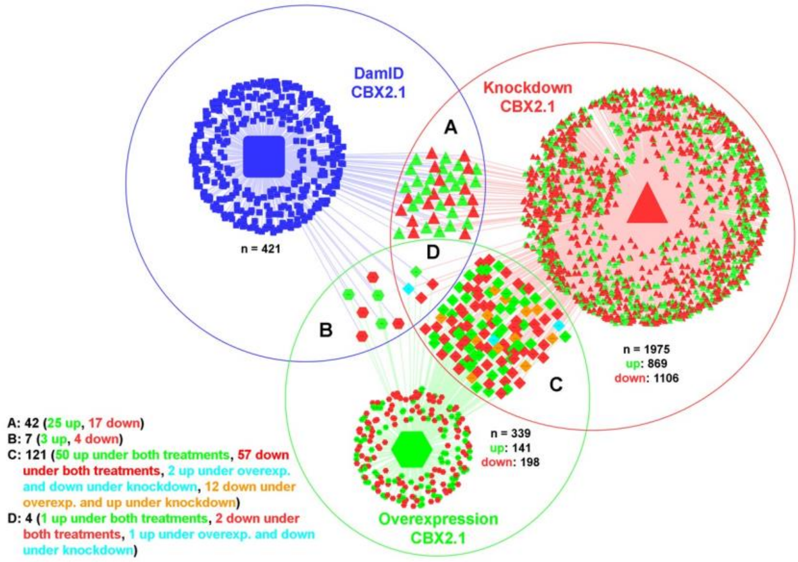

Figure 1. Intersections of ovarian genes regulated by CBX2.1.

A: is the intersection between the DamID-overexpression CBX2.1 genes and RNA seq-knockdown CBX2.1 genes. B: is the intersection between the DamID- overexpression CBX2.1 genes and the RNAseq overexpression CBX2.1 related genes. C: is the intersection between the RNA seq- knockdown CBX2.1 regulated genes and the RNA seq- overexpression CBX2.1 regulated genes. D: is the intersection between A, B and C. We used the false discovery rate as the criterion for determining the set of genes that exhibit differential expression and its critical value has been set to be < 0.05 for all comparisons.

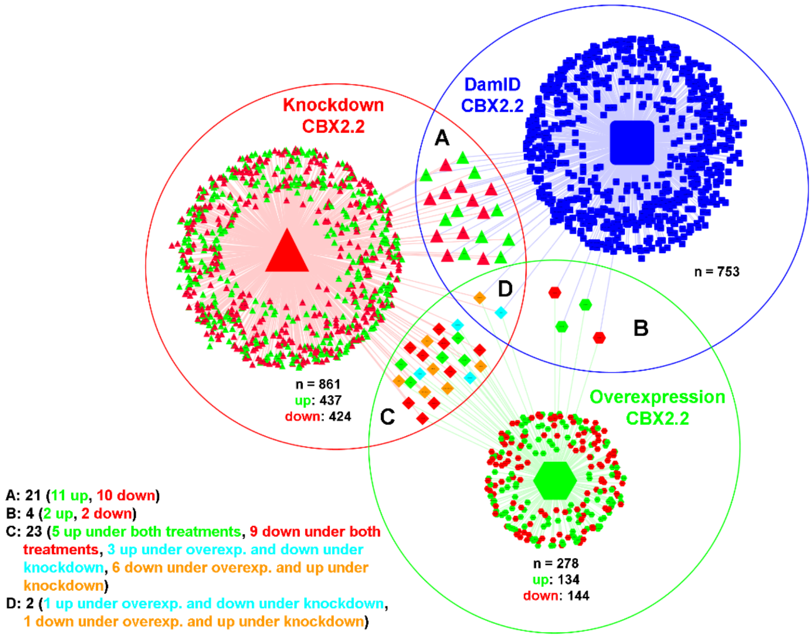

Figure 2. Intersections of ovarian genes regulated by CBX2.2

We used the false discovery rate as the criterion for determining the set of genes that exhibit differential expression and its critical value has been set to 0.05 for all comparisons. A: is the intersection between the DamID-overexpression CBX2.2 genes and RNA seq-knockdown CBX2.2 genes. B: is the intersection between the DamID- overexpression CBX2.2 genes and the RNAseq overexpression CBX2.2 related genes. C: is the intersection between the RNA seq- knockdown CBX2.2 regulated genes and the RNA seq- overexpression CBX2.2 regulated genes. D: is the intersection between A, B and C.

	CBX2.1		CBX2.2	
Gene Name	Fold enrichment	p-value	Fold enrichment	*p*-value
ACTRT1	6.73492	1.27E-10	7.84527	3.54E-06
AGPAT4	4.71444	8.18E-07	4.71266	0.00630812
AKT3	6.73492	1.27E-10	5.24413	0.00196512
ARHGAP36	6.06143	2.64E-09	5.24413	0.00196512
ATP6V1D	3.75359	6.00E-06	5.84503	0.00046323
ATXN1	4.71444	8.18E-07	6.55516	0.00010531
C18ORF10	4.71444	8.18E-07	5.30174	0.00174249
C20ORF144	5.27447	7.98E-08	4.51627	0.00835257
C8B	4.44978	2.45E-06	5.83804	0.00046323
C8ORF48	5.02544	2.21E-07	5.89965	0.00046323
CBX2	7.43178	3.02E-15	6.40969	0.00010531
CD46	5.47608	2.93E-08	5.30174	0.00174249
CRB1	4.47652	2.20E-06	5.89965	0.00046323
DAB1	3.93525	8.44E-06	4.71266	0.00630812
EMBP1	1.58341	8.70E-14	7.05066	2.11E-05
FAM19A4	5.38793	4.92E-08	5.19559	0.00196512
FLJ43390	6.03257	2.30E-09	4.71266	0.00630812
FNDC3B	5.38793	4.92E-08	5.12775	0.00196512
GALC	5.47608	2.93E-08	6.55516	0.00010531
GALNTL6	4.71444	8.18E-07	5.89965	0.00046323
GIGYF1	7.40841	5.59E-12	6.55516	0.00010531
GORAB	5.47608	2.93E-08	4.67102	0.00530958
GOT2	4.71444	8.18E-07	5.89965	0.00046323
GRIK2	4.71444	8.18E-07	5.83804	0.00046323
HACE1	6.06143	2.64E-09	4.40833	0.00835257
HDGFL1	4.82606	4.85E-07	4.67044	0.00630812
HHEX	6.06143	2.64E-09	4.31767	0.01210292
HK2	4.25918	5.18E-06	5.2176	0.00161808
IDS	6.06143	2.64E-09	4.58861	0.00835257
KGFLP1	5.47608	2.93E-08	3.9331	0.03323839
KIAA1210	6.08454	1.87E-09	4.12358	0.0225694
KLF12	5.38793	4.92E-08	6.4799	0.00010531
KLHL4	4.25918	5.18E-06	4.58861	0.00835257
LEPREL1	4.71444	8.18E-07	5.24413	0.00196512
LHCGR	4.71444	8.18E-07	4.58861	0.00835257
LHFP	4.82606	4.85E-07	6.55516	0.00010531
LMO7	7.23909	7.46E-12	6.42185	0.00010531
LMX1A	4.71444	8.18E-07	6.45181	0.00010531
LOC100128505	4.86763	4.12E-07	6.55516	0.00010531
LOC100130000	5.68425	1.30E-09	3.11958	0.03196907
LOC145820	7.40841	5.59E-12	7.21068	2.11E-05
LOC1720	4.25918	5.18E-06	7.21068	2.11E-05

LOC255025	6.03257	2.30E-09	4.90368	0.00343384
LOC283089	5.38793	4.92E-08	6.55516	0.00010531
LOC338864	4.71444	8.18E-07	5.58639	0.00075287
LOC400456	4.25918	5.18E-06	4.12358	0.0225694
LOC440925	6.73492	1.27E-10	6.55516	0.00010531
LOC643486	5.42932	3.52E-08	7.21068	2.11E-05
LOC647012	6.73492	1.27E-10	5.89965	0.00046323
LOC729950	5.75552	9.55E-09	4.58861	0.00835257
LRP1B	4.71444	8.18E-07	5.24413	0.00196512
LRRIQ3	6.06143	2.64E-09	5.89082	0.00046323
LUZP4	3.41235	3.47E-05	5.38224	0.0013736
MAPK8	7.67403	1.28E-12	5.23018	0.00196512
MED30	6.73492	1.27E-10	6.2071	0.00017795
MGMT	5.42932	3.52E-08	6.20065	0.0001208
MIR4500	3.41235	3.47E-05	7.21068	2.11E-05
MIR4671	7.11806	1.20E-11	5.83804	0.00046323
MIR4789	4.71444	8.18E-07	6.55516	0.00010531
MIR708	5.11602	1.53E-07	5.89965	0.00046323
MOCS1	5.38793	4.92E-08	5.89082	0.00046323
NA	1.55635	5.75E-13	1.3826	0.0018999
NAV3	5.38793	4.92E-08	7.21068	2.11E-05
NFIA	5.1381	1.08E-07	5.89082	0.00046323
ODZ4	6.06143	2.64E-09	6.55516	0.00010531
OLFM4	6.08454	1.87E-09	5.89965	0.00046323
OR7E156P	6.11066	2.69E-10	6.42185	0.00010531
PALM2-AKAP2	5.47608	2.93E-08	7.4959	6.49E-06
PCBP3	7.40841	5.59E-12	7.21068	2.11E-05
PCDH9	8.75539	8.37E-15	7.86619	3.54E-06
PCLO	5.38793	4.92E-08	5.89965	0.00046323
PHF3	6.73492	1.27E-10	4.12358	0.0225694
PITX2	4.66734	8.91E-07	6.42185	0.00010531
PLA2G4A	6.06143	2.64E-09	4.12358	0.0225694
PLD5	6.06143	2.64E-09	4.37261	0.01059449
PTGER2	4.86763	4.12E-07	5.24413	0.00196512
PTPRG	4.87654	2.85E-07	5.89965	0.00046323
PURG	6.39503	5.36E-10	5.24413	0.00196512
RGS18	4.71444	8.18E-07	5.30174	0.00174249
ROBO1	4.04095	1.19E-05	4.17517	0.00835257
RPH3A	7.40841	5.59E-12	4.58861	0.00835257
RPL10L	6.06143	2.64E-09	6.55516	0.00010531
SEMA3C	5.38793	4.92E-08	5.51041	0.00072545
SIPA1L2	4.69306	8.08E-07	4.71266	0.00630812
SLC30A8	6.73492	1.27E-10	4.96568	0.00304362
SP3	5.709	8.02E-09	5.30174	0.00174249
STK32B	6.06143	2.64E-09	5.24413	0.00196512
STT3B	4.86763	4.12E-07	5.73131	0.00048519

TIMP3	6.08454	1.87E-09	4.95937	0.00250721
TLE4	5.38793	4.92E-08	7.17633	1.64E-05
TMEM182	5.247	1.32E-08	5.89965	0.00046323
TRDN	7.2524	1.10E-11	6.45181	0.00010531
TSPAN17	4.53097	1.76E-06	4.96568	0.00304362
TSPYL5	3.93525	8.44E-06	4.71266	0.00630812
VENTXP7	5.38793	4.92E-08	4.12358	0.0225694

Table 1. DamID CBX2.1 *Versus* DamID CBX2.2 (95 overlapped targets).

	CBX2.1		*CBX2.2*	
**Gene name**	**Log2**	***p*-Value**	**Log2**	***p*-Value**
SIGLEC9	5.544	4.69E-06	4.636	0.007006
CXCL10	5.391	1.75E-39	2.312	0.01494
FOSB	4.835	1.10E-52	1.453	0.01049
MX1	4.777	1.27E-38	1.334	0.0001695
FGF12	4.676	0.001708	4.344	0.01164
MX2	4.66	5.29E-42	1.736	4.19E-06
TMPRSS7	4.578	0.00102	5.407	0.001568
GPR132	4.444	0.002642	3.769	0.02856
IFIT1	4.253	6.68E-75	1.574	4.37E-09
OAS2	4.209	4.01E-26	1.441	0.0002251
NFATC2	4.195	2.03E-54	1.518	0.004543
TRIM54	4.089	0.005982	3.847	0.03092
TRIM74	4.089	0.005984	3.38	0.04835
PRDM16	4.058	0.009905	3.38	0.04835
ENPP3	3.881	0.01093	4.237	0.01702
RAB40AL	3.88	0.01093	3.654	0.03382
EOMES	3.648	0.02837	4.344	0.01164
EPHX3	3.642	0.02321	3.769	0.02855
ANKRD20A2	3.637	0.02016	3.99	0.01983
ANO1	3.637	0.02017	3.711	0.02886
DUSP26	3.637	0.02016	5.94	0.004941
FAM47C	3.637	0.02016	3.711	0.02886
IFITM1	3.611	7.64E-17	1.057	0.01837
ESM1	3.51	3.84E-24	1.353	0.02046
IFI44L	3.434	2.04E-28	1.344	4.81E-05
BST2	3.416	4.79E-26	1.045	0.0332
LGALS9	3.409	3.28E-30	1.087	0.036
CDHR2	3.341	0.04024	5.571	0.007451
TMEM132B	3.265	5.19E-33	1.194	0.02967
BRINP3	3.248	0.0005058	4.445	0.0003834
DUSP6	3.059	1.15E-65	1.239	4.70E-06
NKD1	2.919	0.000114	3.091	0.01725
IL33	2.88	9.63E-19	2.764	2.40E-12
SP110	2.833	3.64E-15	1.441	0.008575

CXCR4	2.807	2.94E-11	3.366	1.46E-05
FOS	2.802	7.72E-21	1.185	0.03755
HERC5	2.755	2.06E-44	1.058	0.0001109
IFI44	2.748	2.59E-32	1.379	9.61E-07
NAMPT	2.674	5.90E-67	1.27	8.29E-10
CCL20	2.643	8.22E-29	1.081	0.02731
HS3ST3B1	2.634	2.75E-36	1.522	1.87E-06
RGCC	2.542	3.82E-05	2.486	0.01486
LAMP3	2.521	3.14E-18	1.189	0.03062
COL11A1	2.513	0.0002631	-3.325	0.04107
CCND2	2.486	3.37E-20	1.253	0.002253
SLC22A3	2.433	8.38E-27	1.074	0.0009951
BTC	2.425	2.95E-20	1.584	3.89E-05
SERPINB4	2.368	0.03185	4.024	0.00797
PROX1	2.361	1.19E-06	1.889	0.04913
IDO1	2.34	6.23E-05	3.206	0.01496
TMEM200A	2.331	7.81E-31	1.763	1.37E-11
AREG	2.316	3.26E-15	1.486	0.007893
SAMD3	2.288	0.01618	3.255	0.005997
GDNF	2.28	4.67E-32	1.25	8.75E-06
RP11-724O16.1	2.277	0.001163	1.597	0.04382
MAK	2.274	4.47E-05	2.909	0.007892
ZPLD1	2.273	1.25E-16	1.172	0.03726
TSLP	2.158	1.62E-13	1.247	0.02433
SPRY2	2.155	6.43E-27	1.099	0.0002589
SKOR1	2.131	0.02454	2.205	0.04436
CNIH3	2.128	2.26E-20	1.352	0.0004487
LTBP1	2.045	7.07E-24	2.131	6.62E-14
EHF	2.02	3.87E-10	1.585	0.007887
HBEGF	1.983	5.53E-31	-1.083	5.43E-05
KRT34	1.935	9.16E-31	-1.283	0.0001034
KCND2	1.858	1.29E-09	2.035	0.0001901
EMP1	1.82	3.53E-37	1.887	2.31E-25
ISOC1	1.819	2.42E-14	1.646	9.33E-07
PIK3R2	1.776	1.78E-18	2.017	7.23E-23
MYCT1	1.774	8.34E-09	-2.71	0.003365
TNFRSF10B	1.766	4.97E-43	1.006	3.63E-11
SLC7A11	1.739	7.51E-22	-1.132	2.96E-07
PHLDA1	1.731	1.33E-26	1.422	3.55E-13
DUSP4	1.709	1.10E-25	1.064	1.26E-07
RNASE7	1.701	0.0162	-3.682	0.02187
HAS2	1.685	9.56E-17	1.26	8.04E-08
AC013271.3	1.643	0.03615	4.279	0.003955
FAM155A	1.641	5.34E-12	1.401	1.92E-05
LINGO2	1.625	0.0001071	1.619	0.04238
KYNU	1.618	1.09E-21	1.156	1.46E-07

SAT1	1.602	1.97E-27	1.324	1.55E-10
GLDN	1.58	0.0003611	1.754	0.03532
OPN3	1.572	3.30E-18	1.494	5.41E-11
CCDC65	1.53	0.03252	2.946	0.007377
CXCL3	1.516	2.88E-16	1.248	1.52E-08
RAB27B	1.501	9.94E-18	1.813	1.04E-15
DUSP2	1.497	0.009574	-4.777	0.001346
CD44	1.477	2.38E-25	1.19	6.38E-11
KIAA1211	1.452	3.93E-06	1.712	0.0005445
PDE2A	1.45	1.86E-07	1.074	0.003094
LRRC6	1.448	4.47E-05	1.865	0.004529
KCNK13	1.443	0.0008147	-2.777	0.01522
SIGLEC15	1.443	1.73E-05	1.461	0.0188
CD22	1.438	1.51E-07	1.813	6.81E-05
NCOA7	1.406	3.62E-16	1.063	1.13E-06
GBP5	1.405	1.98E-07	1.234	0.0008812
RORA	1.385	7.66E-08	1.041	0.01541
KCNH1	1.358	2.18E-07	-1.899	0.0007923
PCDH12	1.358	6.92E-05	1.445	0.03194
SH2D5	1.353	8.64E-10	-1.023	0.009606
FOPNL	1.35	4.44E-14	1.569	1.35E-12
NLRP10	1.341	0.0005985	-3.546	0.0003808
SLC4A10	1.323	0.0002773	2.216	0.0006303
NDRG1	1.301	3.51E-15	1.184	3.62E-07
ZNF844	1.278	4.98E-06	1.317	0.01644
TGFBR3	1.276	1.97E-10	1.038	0.0002041
PCTP	1.248	3.09E-11	1.082	1.50E-05
TNFSF4	1.235	2.34E-06	1.487	1.26E-05
CSF2RA	1.227	3.97E-08	-1.254	0.04711
RGPD6	1.204	7.27E-13	1.081	7.42E-06
FAM81A	1.191	1.25E-05	1.472	0.0009289
KIAA0408	1.175	0.002405	1.107	0.04271
PPARGC1A	1.167	3.06E-09	-1.092	0.003656
SOGA3	1.157	0.004956	2.595	0.001124
CHST3	1.154	2.05E-17	1.259	2.89E-14
CTD-2207O23.12	1.154	0.004849	1.618	0.02563
CHAC1	1.143	1.49E-05	-2.132	2.23E-07
GALNT3	1.132	2.78E-08	-1.109	0.001451
PTGES	1.114	0.0007451	1.708	0.00021
SLC7A5	1.114	1.71E-06	-1.44	4.83E-07
ARRDC3	1.108	3.20E-11	1.077	3.38E-06
PIWIL4	1.108	0.0001588	1.416	0.009216
DDIT4L	1.106	7.68E-06	1.07	0.002823
HSD11B1	1.098	2.44E-05	1.241	0.0004091
ITGA2	1.085	4.96E-11	1.193	2.51E-09
AC015688.3	1.08	0.01597	2.208	0.03623

CDK6	1.078	2.84E-14	-1.111	1.95E-08
TMEM65	1.074	1.48E-07	1.31	5.93E-07
SLC2A13	1.051	9.62E-08	1.126	3.69E-06
TNFAIP6	1.05	0.0002461	1.457	0.00156
DPY19L1	1.04	5.79E-15	1.062	1.13E-08
P2RY1	1.031	3.92E-06	1.02	0.003589
RNF170	1.029	1.86E-09	1.264	5.06E-07
B3GNT5	1.023	1.58E-05	1.317	0.0003728
SHC4	1.022	0.000201	1.245	0.01218
THBS1	-1.004	1.20E-13	-1.38	4.93E-12
CORO6	-1.011	0.005135	1.226	0.02191
CASZ1	-1.024	0.001182	-1.906	0.0005217
GALNT15	-1.032	0.0003089	1.068	0.006184
HSPG2	-1.049	0.001389	-1.002	0.006864
MGAM	-1.053	2.07E-13	-1.472	1.19E-10
ACPP	-1.069	0.01034	1.345	0.03652
PMF1-BGLAP	-1.072	0.001819	-1.362	0.03465
SEMA4G	-1.079	8.34E-05	1.22	4.47E-06
SCN1B	-1.094	0.0005448	1.19	0.0004012
TMEM184A	-1.105	0.000546	-1.29	0.006516
TSPAN8	-1.117	5.14E-06	1.012	0.001411
YPEL1	-1.13	0.001324	1.818	0.0002877
NOX5	-1.136	0.0003266	1.456	0.0004475
HUNK	-1.141	4.84E-06	-1.201	0.001618
CDC42EP1	-1.16	5.15E-05	-1.154	0.0007594
CTSV	-1.162	6.26E-05	1.013	0.003921
ABCG4	-1.168	0.002604	1.236	0.0392
VASH2	-1.175	0.0006663	1.746	0.0003341
ARNT2	-1.192	9.81E-19	-1.41	5.49E-17
SERHL2	-1.208	0.0004099	1.225	0.01559
CTB-55O6.8	-1.21	0.01247	1.323	0.04772
C10orf54	-1.213	1.01E-07	-1.289	9.91E-08
FLNA	-1.233	1.44E-06	-1.099	0.0001064
COL5A1	-1.238	4.82E-11	-1.027	3.64E-06
ST6GALNAC2	-1.238	0.007562	1.967	0.001254
KCNMB4	-1.242	9.62E-05	1.196	0.009361
ELFN2	-1.246	3.63E-06	-1.27	0.0004866
CNN2	-1.248	1.98E-12	-1.143	2.97E-07
MT1F	-1.249	7.70E-06	-1.181	0.001914
ZNF385B	-1.252	0.001983	1.67	0.01142
ST6GALNAC5	-1.273	8.49E-15	-1.137	4.08E-08
TCF7	-1.283	0.001516	1.625	0.0003737
ZCCHC5	-1.285	1.41E-05	-1.506	0.003727
LRP5	-1.31	2.93E-05	-1.115	0.003333
KRT86	-1.311	3.79E-06	-1.218	0.01236
TM4SF18	-1.314	0.000476	1.478	0.01304

PDLIM7	-1.317	4.47E-08	-1.056	5.38E-05
NME2	-1.326	2.92E-08	-1.09	0.001009
NPPB	-1.327	0.00186	-3.412	0.0005131
AP1M2	-1.334	1.11E-08	-1.574	1.55E-05
CXCL6	-1.342	8.16E-09	1.384	2.29E-07
MMP24	-1.343	4.42E-13	-1.068	1.25E-05
HTR1B	-1.352	0.02162	-2.734	0.009903
ABCA6	-1.382	0.0009823	1.454	0.02836
RDH5	-1.406	2.40E-07	-1.075	0.01181
NPB	-1.433	0.01085	-2.241	0.02491
NGEF	-1.479	7.29E-07	1.623	1.11E-06
TIAF1	-1.48	1.47E-09	-1.151	0.009215
EPHB3	-1.491	0.001507	1.29	0.04932
CYS1	-1.513	0.008453	1.537	0.03524
HSPB7	-1.518	1.69E-15	-1.277	4.55E-08
TLR7	-1.533	0.02233	-3.579	0.002764
FGF18	-1.542	0.03337	-3.345	0.006421
NUGGC	-1.542	0.03432	-2.523	0.02356
POTEE	-1.57	2.31E-07	-1.507	0.01499
LIMS2	-1.597	2.31E-08	-1.159	0.00718
KCNAB1	-1.617	0.003027	-2.243	0.02502
ACTB	-1.62	1.29E-22	-1.307	1.06E-08
CRB2	-1.62	0.01625	-2.096	0.04385
RDM1	-1.642	0.0005524	1.818	0.002062
MYPN	-1.645	1.44E-12	-1.286	0.0001041
PSG8	-1.651	0.004041	-2.633	0.007901
ERBB3	-1.665	1.83E-08	-1	0.005128
SYT2	-1.678	0.0459	-3.225	0.008318
VAT1L	-1.684	9.87E-05	1.33	0.02215
PROCA1	-1.713	3.06E-05	1.322	0.02134
RN7SL1	-1.725	4.94E-05	-1.404	0.0008274
EFHD1	-1.726	0.001129	1.686	0.02111
FSCN1	-1.734	8.03E-11	-1.775	2.22E-07
IER5L	-1.815	1.11E-08	-1.005	0.003471
PLCD4	-1.826	6.00E-06	1.775	0.0003338
GFRA1	-1.841	5.42E-20	-1.055	2.34E-06
RP11-1035H13.3	-1.87	0.0006561	-2.543	0.0001317
DIO2	-1.884	1.08E-13	-1.838	9.15E-09
ID3	-1.892	2.80E-14	-1.04	0.0006341
HSPB1	-1.894	1.12E-09	-1.168	0.007668
DOC2A	-1.908	1.26E-07	1.047	0.03668
RORC	-1.922	0.02064	2.328	0.04545
COL1A1	-1.924	3.77E-14	-1.352	4.91E-05
CHRNA6	-1.93	0.04112	-2.71	0.04743
CTAGE1	-1.939	0.004858	-3.076	0.005336
SYT15	-1.94	1.17E-19	-1.582	0.003457

MYL9	-1.941	1.41E-21	-1.465	3.09E-11
SERPINA6	-1.946	3.67E-08	1.375	0.003493
RP11-548K23.11	-1.953	4.21E-08	-1.166	0.03325
GPRC5C	-1.957	3.82E-06	-1.393	0.04438
MEST	-1.99	6.65E-33	-1.168	5.54E-10
CHST4	-2.014	0.0003069	-2.442	0.01291
HSPB3	-2.041	8.23E-10	-1.255	0.006432
NPTX1	-2.061	0.01226	-4.805	0.000145
DQX1	-2.062	0.03762	2.699	0.02286
RAB26	-2.063	8.37E-05	1.64	0.007935
PCSK1N	-2.067	0.02771	-2.707	0.0341
PLN	-2.073	0.03761	-2.756	0.03571
ADGRG5	-2.083	0.04499	2.045	0.0296
ADAMTS15	-2.088	3.74E-15	-1.636	0.04124
ACKR3	-2.092	4.38E-10	-2.199	0.006931
COL21A1	-2.11	0.001133	1.638	0.03519
RNA5-8S5	-2.115	0.02804	-4.375	0.0002248
TAGLN	-2.144	5.11E-34	-2.057	3.85E-19
CD24	-2.198	4.27E-14	-1.141	0.002464
ZMYND10	-2.213	6.03E-07	1.412	0.01507
TRPC3	-2.239	4.35E-08	1.216	0.03472
CACNG4	-2.285	9.13E-16	-1.219	0.0002786
C2orf15	-2.303	0.005519	-3.362	0.008417
MRVI1	-2.316	4.59E-14	-1.155	0.003098
OLR1	-2.372	0.04114	-3.991	0.01215
CEMIP	-2.376	1.90E-56	-1.024	1.22E-09
TRIM29	-2.396	1.19E-15	-1.208	0.00435
MYO18B	-2.436	0.0014	-2.782	0.01042
ALS2CR11	-2.447	0.0003313	-3.225	0.003294
SRL	-2.488	0.01469	-3.135	0.02151
AC104389.28	-2.52	3.89E-11	-1.29	0.042
PLEKHA7	-2.539	5.24E-07	-2.86	0.004365
PCP4L1	-2.544	0.00105	-3.679	0.002714
DEPTOR	-2.597	1.35E-23	-1.072	0.0001847
FAM83C	-2.617	0.01583	-2.507	0.04777
RTP3	-2.617	0.01584	-4.233	0.005304
C4BPA	-2.618	0.01605	-4.255	0.005347
ATOH8	-2.701	5.27E-32	-1.378	5.48E-06
CNN1	-2.704	4.37E-36	-2.491	2.35E-14
B3GALT2	-2.733	0.0003185	-2.602	0.01401
CGB5	-2.798	2.13E-13	-2.451	0.0037
LDB3	-2.862	2.94E-12	-2.763	0.001032
SOAT2	-2.896	3.63E-05	-3.228	0.002104
DOK7	-2.93	0.0003883	-2.843	0.01093
EDN2	-3.07	2.62E-15	-2.021	0.01984
ELF3	-3.102	1.41E-17	-1.065	0.03898

METTL7A	-3.307	4.02E-26	1.26	5.87E-05
HIST1H3C	-3.316	0.0009261	-3.113	0.01448
ACTA2	-3.352	7.80E-37	-2.881	6.12E-24
SLC34A3	-3.399	0.02375	-3.295	0.04252
C9orf92	-3.404	0.02358	-3.322	0.04124
CLCNKB	-3.404	0.02358	-3.322	0.04124
KRT6B	-3.404	0.02358	-3.322	0.04124
AC005785.2	-3.405	0.02356	-3.325	0.04107
LCT	-3.405	0.02356	-3.325	0.04107
HIST1H2BL	-3.406	0.02354	-3.329	0.04091
RP11-420N3.3	-3.406	0.02354	-3.329	0.04091
TRBV14	-3.41	0.02339	-3.352	0.0398
DTX1	-3.411	0.02337	-3.356	0.03964
GSTA2	-3.413	9.18E-13	-3.172	0.0003812
EPPK1	-3.551	4.77E-16	-2.856	0.001095
LGI1	-3.701	6.96E-05	-2.862	0.01174
KLHDC8A	-3.716	1.16E-09	1.499	0.02878
LEP	-3.788	0.009128	-3.716	0.02086
APOH	-3.792	0.009058	-3.737	0.02024
MAP2K6	-3.959	8.82E-13	1.767	0.001741
CLIC3	-4.025	5.47E-21	-1.345	0.02839
ACTG2	-4.076	8.90E-33	-3.627	3.89E-19
LMOD1	-4.22	3.83E-66	-2.067	2.77E-16
GSTA1	-4.471	1.35E-21	-3.349	6.54E-05
PLA2G5	-4.552	0.0005553	-2.756	0.03374
SOST	-5.158	3.39E-05	-3.221	0.01096
ITIH3	-5.502	5.21E-06	-2.75	0.02556
ERMN	-6.692	1.18E-12	-2.264	0.01634
CBX2	9.212	1.93E-59	7.006	6.59E-74
CACNA2D2	4.203	0.005571	3.823	0.009635
ITGAL	4.203	0.005571	3.152	0.04812
CTD-2368P22.1	4.174	0.005136	4.766	0.0003349
TBPL2	3.958	0.00813	3.536	0.02299
RASSF10	3.955	0.007688	3.152	0.04812
NTRK1	3.789	0.03307	3.159	0.0477
EPHB6	3.726	0.01686	3.152	0.04812
ANKRD35	3.721	0.01552	3.152	0.04812
LST1	3.712	0.01482	4.067	0.004603
DRD5	3.429	0.03271	3.145	0.04853
C9orf66	3.429	0.03271	3.823	0.009643
UCA1	3.419	0.03319	3.536	0.02299
RP11-53I6.2	3.412	0.03352	3.152	0.04812
ANGPTL4	3.349	1.81E-24	1.758	0.000581
CACNG2	3.328	0.0005074	3.015	0.003365
SPNS3	2.526	0.01771	2.882	0.006188
GRIA2	2.511	0.02074	2.368	0.046

C3orf70	2.408	0.01089	2.112	0.03453
PSMA1	2.349	2.49E-07	1.557	0.001565
C11orf96	2.174	0.004845	1.848	0.03809
WISP2	2.171	0.02766	2.484	0.01417
FCER1G	2.169	0.00455	2.233	0.006786
CR1L	2.1	0.01268	1.947	0.01865
ZFP92	1.983	0.0003074	1.278	0.04492
SHANK1	1.966	0.0433	2.167	0.004637
IL20	1.907	0.0194	1.569	0.04919
HSFX1	1.809	0.003687	1.277	0.04746
PCDHB7	1.654	0.009733	1.41	0.04346
POC1B-GALNT4	1.443	0.0007195	1.037	0.004465
ZBTB20	1.277	0.005525	1.017	0.02089
AC092835.2	1.133	0.04598	1.138	0.04548
LTF	-1.05	0.0449	-1.383	0.01431
AMY2B	-1.169	0.002614	-1.234	0.0039
PPP1R32	-1.211	0.02267	-1.189	0.02887
CNKSR1	-1.214	0.04183	-1.832	0.00408
C2orf82	-1.322	0.01063	-1.379	0.01419
KIAA1211L	-1.448	0.002013	-1.449	0.003211
TNFRSF13C	-1.376	0.01473	-1.371	0.02129
FAM46B	-1.57	0.002611	-1.056	0.03559
GUCY1A3	-1.597	0.02426	-1.653	0.0273
SMIM6	-1.636	0.04648	-2.284	0.01662
TNNC2	-1.707	0.04362	-2.053	0.03153
KIAA1755	-1.764	0.004449	-1.995	0.002455
PSG3	-1.77	0.003053	-1.256	0.02884
SCIN	-1.821	0.0007857	-1.442	0.004811
WNT10A	-1.883	8.99E-05	-1.272	0.00302
LINC00173	-1.9	0.04774	-2.456	0.04463
TPBGL	-2.022	0.01396	-2.666	0.02642
UPB1	-2.079	0.02591	-3.902	0.01383
KRT38	-2.197	0.000145	-1.06	0.0372
FAM9C	-2.256	0.01471	-2.013	0.0328
PGBD3	-2.32	0.0002951	-1.892	0.001937
PCSK9	-2.797	0.02101	-3.872	0.01394
CTD-2510F5.6	-2.825	0.001167	-2.076	0.01147
RNA5-8S5	-2.996	0.00016	-2.141	0.01783
SLC6A7	-3.299	0.03241	-2.948	0.006268
OCM	-3.299	0.03241	-3.08	0.002503
CD7	-3.301	0.03233	-3.176	0.04435
SYNDIG1	-3.301	0.03233	-3.18	0.04411
PPP1R1B	-3.301	0.03233	-3.185	0.04385
FGF17	-3.31	0.03196	-3.185	0.04387
C1orf234	-3.31	0.03196	-3.19	0.04361
LRP2	-3.31	0.03196	-3.19	0.04361

HIST1H2BB	-3.312	0.03189	-3.19	0.04361
ANO1	-3.312	0.03189	-3.194	0.04337
CALR3	-3.455	0.001213	-3.56	0.02072
HSD17B2	-3.68	0.01406	-3.556	0.02086
TMED6	-3.692	0.01378	-3.565	0.02057
LCK	-3.835	1.33E-05	-3.862	0.009168
ZNF423	-3.984	0.005479	-3.869	0.009268
HIST1H3C	-4.24	0.002407	-2.67	0.02694
TMC5	-4.244	0.003536	-3.569	0.02043
AC003002.6	-2.634	1.78E-06	1.333	0.04371
ACTC1	-2.283	5.04E-07	-2.485	0.00128
AMHR2	-4.545	0.0005025	-1.814	0.04454
ANGPTL4	3.548	2.12E-31	1.758	0.000581
ANO1	3.637	0.02017	-3.194	0.04337
AWAT2	-2.809	0.00292	-1.648	0.02135
C11orf96	1.683	0.02472	1.848	0.03809
C2orf82	1.26	0.01949	-1.379	0.01419
C4orf48	-1.7	8.62E-05	1.883	0.02336
CBX2	-3.369	5.17E-25	7.006	6.59E-74
CELA1	-3.782	0.009228	-3.56	0.02072
CR1L	1.778	0.03333	1.947	0.01865
DNAJC15	-1.383	3.14E-05	-1.001	0.02711
DRD5	3.884	0.0113	3.145	0.04853
EFHB	-1.696	0.03258	-3.19	0.04361
FAM25A	-3.405	0.02356	-3.862	0.008198
FAM46B	-1.666	0.000135	-1.056	0.03559
FAM47C	3.637	0.02016	-3.185	0.04387
FCER1G	1.988	0.0404	2.233	0.006786
GPBAR1	2.202	0.04275	-3.56	0.02072
GRIN1	-3.404	0.02358	3.159	0.0477
GSTA1	-4.471	1.35E-21	-1.462	0.04478
HIST1H3C	-3.316	0.0009261	-2.67	0.02694
HRCT1	-1.493	0.001976	-1.379	0.005124
HSFX1	1.327	0.02097	1.277	0.04746
IL20	2.458	0.000405	1.569	0.04919
KCNA4	-3.014	0.003053	-2.638	0.02795
KIAA1755	1.926	0.0007454	-1.995	0.002455
KIF26A	-2.617	0.01584	2.192	0.01322
KREMEN2	-4.085	0.003122	2.423	0.04654
KRT38	-1.846	0.002171	-1.06	0.0372
LAMA5	-1.649	5.09E-07	1.019	0.03744
LDLRAD2	-1.321	0.03787	1.582	0.0455
LGI3	-1.343	0.0134	-1.396	0.03242
LINC00173	-2.072	0.03009	-2.456	0.04463
LYL1	-1.512	0.007228	1.547	0.03294
MS4A4E	-1.085	0.02469	1.65	0.03346

NYNRIN	-2.406	0.0001365	-3.194	0.04337
PCED1B-AS1	-2.416	0.007799	-3.858	0.01091
PCSK1N	-2.067	0.02771	2.859	0.002868
POTEE	-1.57	2.31E-07	-1.397	0.01589
PRKCG	-2.379	0.04494	4.067	0.004605
PSG3	-1.962	0.001165	-1.256	0.02884
RIPPLY1	3.637	0.02016	-1.794	0.03503
RNA5-8S5	-2.115	0.02804	-2.141	0.01783
RNASE7	1.701	0.0162	-2.896	0.002375
RP11-5A19.5	-1.702	0.004509	1.521	0.01883
RP11-795F19.5	2.201	0.0456	-1.858	0.0493
SCIN	-2.308	8.93E-10	-1.442	0.004811
SCN2B	-3.015	0.003471	3.145	0.04853
SHANK1	1.488	0.004142	2.167	0.004637
SLC35F3	3.352	0.03966	-3.547	0.02114
SLC39A12	-2.374	0.03775	-3.135	0.01599
SLC8A2	2.191	0.04975	3.152	0.04811
SMIM6	-5.515	7.67E-07	-2.284	0.01662
SPANXN4	3.883	0.01119	-3.56	0.02073
SULT2B1	-2.368	0.03988	-3.19	0.04361
TMPRSS7	4.578	0.00102	-2.45	0.0288
TNFRSF13C	-1.198	0.01294	-1.371	0.02129
TUBB3	-2.183	1.85E-15	1.682	0.0055
UNC13A	1.833	0.01631	-3.858	0.01092
UPB1	-2.572	1.38E-05	-3.902	0.01383
WISP2	-1.531	0.02175	2.484	0.01417
WNT10A	-2.737	2.43E-09	-1.272	0.00302
ZFP92	2.039	0.0001629	1.278	0.04492
ZNF423	-5.941	1.59E-07	-3.869	0.009268
AC14013271.3	-1.648	0.03464	4.279	0.003955
AC084219.2	1.716	0.001926	1.503	0.005737
ACTA2	-1.296	1.50E-07	-2.881	6.12E-24
ADGRG2	-2.853	0.009077	-2.875	0.02387
AMY2B	-1.169	0.002614	1.153	0.02888
ANO1	-3.312	0.03189	3.711	0.02886
ARL14	-1.771	0.01926	2.744	0.01064
ATOH8	-1.071	0.009478	-1.378	5.48E-06
C4BPA	-3.685	0.01394	-4.255	0.005347
C5orf46	-1.321	0.01304	-2.044	0.03887
COL1A1	-1.232	0.0005872	-1.352	4.91E-05
COL21A1	-1.538	0.01212	1.638	0.03519
CRB2	-4.234	0.001967	-2.096	0.04385
CXCR4	1.343	0.0003691	3.366	1.46E-05
DNAH10	4.156	0.006781	-4.018	0.009874
DOK7	-2.431	0.04148	-2.843	0.01093
ELF3	-1.976	0.0008051	-1.065	0.03898

ERMN	-1.53	0.02089	-2.264	0.01634
ESM1	1.448	2.83E-07	1.353	0.02046
FAM26D	3.387	0.03467	-3.596	0.003004
FCGR2A	-1.2	0.006833	2.15	0.003694
FGF18	-1.763	0.04242	-3.345	0.006421
FOS	1.382	1.55E-05	1.185	0.03755
GPR132	-3.17	6.85E-05	3.769	0.02856
HIST1H3C	-4.24	0.002407	-3.113	0.01448
HSPB3	-1.19	0.00201	-1.255	0.006432
IL33	1.223	5.39E-06	2.764	2.40E-12
KLHDC8A	-1.088	0.02374	1.499	0.02878
LMOD1	-1.06	0.0003172	-2.067	2.77E-16
LRRC4	2.059	0.03185	-3.325	0.04107
MRVI1	-1.001	0.002205	-1.155	0.003098
NEUROD1	3.956	0.007531	-4.25	0.005348
NKD1	-1.713	0.01058	3.091	0.01725
NLRP10	1.224	0.04764	-3.546	0.0003808
PGBD3	-2.32	0.0002951	-1.839	0.04387
PIK3C2G	3.419	0.03319	-3.329	0.04091
PTGES3L	-1.058	0.03829	-1.904	0.04908
RAB40AL	3.394	0.03434	3.654	0.03382
RN7SL1	-1.204	0.01714	-1.404	0.0008274
RNA5-8S5	-2.996	0.00016	-4.375	0.0002248
RORA	1.099	0.0009858	1.041	0.01541
RP11-53I6.2	3.412	0.03352	-3.359	0.03948
SERPINB4	-1.637	0.04107	4.024	0.00797
SLC22A13	2.375	0.03352	3.246	0.01893
SLC35F4	-3.31	0.03196	3.878	0.02579
SOGA3	1.008	0.02311	2.595	0.001124
SOX3	-2.458	0.03017	-3.133	0.02802
SRL	-3.675	0.01417	-3.135	0.02151
TMEM132B	1.361	0.000269	1.194	0.02967
TRIM29	-1.055	0.011	-1.208	0.00435

Table 2. 481 overlapped targets: RNA seq *CBX2.1 Versus* RNA seq *CBX2.2*
